# Variant-divergent death: Omicron intensifies bystander T-cell apoptosis via GDF15–BCL2L13

**DOI:** 10.1038/s41420-026-03079-x

**Published:** 2026-03-28

**Authors:** Chao Gao, Hanbing Chen, Ying Chi, Xinxing Lu, Jiahuang Li, Ying Tang, Ruixuan Yu, Nan Shi, Ling Liu, Jianfeng Xie, Haibo Qiu, Jie Chao, Shufeng Li

**Affiliations:** 1 Jiangsu Provincial Key Laboratory of Critical Care Medicine, Department of Critical Care Medicine, Zhongda Hospital, School of Medicine, Southeast University, Nanjing, China; 2 Jiangsu Provincial Center for Disease Control and Prevention, NHC Key laboratory of Enteric Pathogenic Microbiology, Nanjing, China; 3 Department of Critical Care Medicine, Taizhou School of Clinical Medicine, The Affiliated Taizhou People’s Hospital of Nanjing Medical University, Taizhou, China; 4 School of Biopharmacy, China Pharmaceutical University, Nanjing, China; 5 Department of Physiology, School of Medicine, Southeast University, Nanjing, China; 6 Department of Biochemistry, School of Medicine, Southeast University, Nanjing, China

**Keywords:** Viral infection, Infection

## Abstract

**Background:**

Severe Omicron cases present profound lymphocytopenia, suggesting variant-specific immune injury.

**Results:**

We identify CD63 as a conserved T-cell host factor supporting ACE2-independent SARS-CoV-2 entry. Despite lower intracellular viral loads than the ancestral strain, Omicron elicits enhanced T-cell apoptosis largely through a bystander mechanism. Omicron-stimulated epithelial cells secrete GDF15, which upregulates the pro-apoptotic protein BCL2L13 in T cells and thereby remotely accelerates apoptosis in uninfected bystanders. Functionally, recombinant GDF15 increases BCL2L13 and apoptosis, while genetic dampening of BCL2L13 blunts Omicron-specific high-intensity bystander death. In clinical samples, plasma GDF15 associates with mortality, SOFA scores, and lower lymphocyte counts, bridging the epithelial–immune axis to patient outcomes.

**Conclusions:**

Our data delineate a two-track model of Omicron immune injury—CD63-enabled T-cell entry plus GDF15–BCL2L13-driven bystander apoptosis—that reconciles lower epithelial cytopathicity with deeper T-cell depletion in critical disease. These findings nominate the GDF15–BCL2L13 axis as a mechanistic marker and potential point of intervention.

## Introduction

Lymphocytopenia—most prominently affecting T cells—is a hallmark of COVID-19 immunopathology and correlates with poor outcomes in critical illness [1–4]. Clinical cohorts have further noted that patients infected during the Omicron-dominant period often present with lower peripheral T-cell counts than those infected earlier [5], raising the possibility that Omicron imposes a distinct pattern of immune injury. Clarifying why T-cell depletion is accentuated in these patients is important for understanding immune dysregulation in severe disease and for identifying tractable therapeutic targets.

Canonically, SARS-CoV-2 enters host cells through Spike–ACE2 engagement [6, 7], yet this model alone does not explain viral signals detected in T lymphocytes, which express little to no ACE2. Several host factors, including CD147 [8] and neuropilin-1 [9], have been proposed to assist Spike-mediated entry, suggesting that multiple entry routes may expand viral cellular tropism across microenvironments. Whether and how these ACE2-independent mechanisms operate in T cells, and whether they differ between Omicron and the ancestral strain, remain open questions. Beyond direct infection, virus-induced “bystander apoptosis”—well documented in other viral infections—could further deplete uninfected T cells [10, 11], but its occurrence and molecular drivers in COVID-19 are not established.

Here, we test the hypothesis that variant-conserved entry coexists with variant-divergent death in T cells: (i) a conserved host factor mediates ACE2-independent entry into T cells; and (ii) Omicron accentuates T-cell loss by potentiating bystander apoptosis through epithelial - immune crosstalk. This framework aims to reconcile clinical lymphocytopenia with mechanistic underpinnings and to nominate targets for mitigating immune exhaustion in critical COVID-19. This two-track model—conserved entry and divergent execution—provides a mechanistic bridge between lower epithelial cytopathicity and deeper T-cell loss in severe Omicron disease.

## Discussion

This study connects variant-conserved entry to variant-divergent death in T cells. We show that CD63, a tetraspanin linked to viral entry platforms, functions as a conserved host factor for SARS-CoV-2 entry into T cells, supporting infection independently of ACE2. At the same time, Omicron engages an epithelial–immune crosstalk that releases GDF15, which elevates BCL2L13 in T cells and amplifies bystander apoptosis. This two-track paradigm explains how Omicron can exhibit reduced epithelial cytopathicity yet drive deeper lymphocyte loss in critical illness, providing a mechanistic bridge from cell-intrinsic entry to paracrine execution of T-cell death.

CD63, a tetraspanin known to be involved in organizing membrane microdomains that can serve as viral entry platforms, has previously been reported in association with infections by HIV [12–14], Lujo virus [15], and White Spot Syndrome Virus [16]; we demonstrated the role of CD63 in SARS-CoV-2 infection of T cells. CD63-mediated SARS-CoV-2 infection is enhanced under low pH conditions, which helps explain the correlation between COVID-19 severity and blood pH in patients[17]. Additionally, we found that viral load in T cells infected with Omicron was lower than that in the ancestral strain group, contrasting with their respective tropisms for respiratory epithelial cells, this observation suggests that during SARS-CoV-2 evolution, a “cell type-dependent infection strategy” is employed to balance transmissibility and immune evasion capabilities: high affinity for epithelial cells enhances transmission, while low-level replication in T cells facilitates immune surveillance evasion.

Unexpectedly, it was also discovered that Omicron infection induces enhanced T-cell apoptosis via a “bystander effect”, potentially explaining the low lymphocyte and T-cell counts observed in Omicron-infected individuals [5, 18]. It is important to note that the Omicron cohort’s older age and higher comorbidity burden largely accounted for the lower average lymphocyte counts on initial comparison. Our adjusted analyses confirmed that differences in mean lymphocyte levels between Omicron and ancestral severe cases could be explained by these baseline factors. However, even after controlling for confounders, Omicron patients remained overrepresented at the extreme low end of the lymphocyte distribution. In practice, this means a subset of Omicron-infected ICU patients experienced profoundly lower T-cell counts than expected, aligning with the bystander T-cell apoptosis mechanism. Although this lower-tail difference did not reach statistical significance, the consistent trend supports our proposed GDF15–BCL2L13 “apoptosis amplifier” model. From a clinical perspective, this finding underscores that Omicron’s impact on immune dysregulation may manifest not as a shift in average lymphocyte count, but as an increased risk of critical lymphocyte depletion in vulnerable individuals—a nuance that helps explain the pronounced T-cell apoptosis and immune injury observed in severe Omicron infections.

Collectively, bystander T cell apoptosis implies that Omicron may cause widespread T-cell destruction extending beyond directly infected cells, possibly mediated by soluble factors released from infected or other activated cells, thereby significantly contributing to immune dysregulation. An innovative mechanism is proposed wherein the GDF15-BCL2L13 axis amplifies this bystander T-cell apoptosis. This axis may provide key molecular markers for characterizing features of immune exhaustion and prognostic differences in individuals infected with Omicron. GDF15 levels in male plasma are higher than in female plasma, providing a potential explanation for the clinical observation of a higher proportion of male severe Omicron cases. Furthermore, this proposed pathway offers an important theoretical basis for understanding the clinical paradox where Omicron infection generally has a lower rate of severe disease progression, yet severe patients can exhibit more profound immune injury, thus contributing to the observed clinical heterogeneity. Notably, multiple mutation sites within the Omicron Spike protein (e.g., K417N, E484A) may enhance its binding efficiency to host cell surface receptors (such as ACE2) [19]. This enhanced interaction may, in turn, amplify GDF15 secretion signals, providing structural biology clues that help explain the stronger pro-apoptotic effect of Omicron compared to the ancestral strain. This suggests a cascade where viral genetic evolution directly influences host cytokine responses and subsequent immunopathology.

This study has several limitations. First, we focused on infections by the widespread Omicron variant versus the ancestral SARS-CoV-2 strain to interrogate conservation and divergence of T-cell infection across evolution; intermediate variants such as Delta were not included, and future work incorporating these variants could better resolve the evolutionary trajectory of T-cell interactions. Second, clinical cohort sizes and single-cell sequencing datasets were relatively small, reflecting the sampling window during the pandemic. Although we integrated public resources to enhance generalizability, the inherent constraints of smaller primary datasets—limited statistical power and robustness—remain. Third, the mechanisms of bystander T-cell apoptosis were interrogated mainly in vitro. While a biomimetic lung microphysiological system reproducing the alveolar air–liquid interface provided greater physiological relevance than conventional cultures, appropriate in vivo models will be required to capture systemic complexity and validate these mechanisms in whole organisms.

Together, our data position GDF15–BCL2L13 as an actionable axis that marks and magnifies bystander T-cell apoptosis during Omicron infection, with plasma GDF15 tracking severity and lymphocyte depletion. While the precise upstream receptor(s) on T cells remain to be defined, the functional linkage between epithelial GDF15 and T-cell BCL2L13 offers testable paths for risk stratification and intervention. We therefore propose a working model in which CD63-enabled entry seeds infection within T cells, whereas GDF15-driven BCL2L13 induction propagates apoptosis among uninfected bystanders—together shaping the immune landscape of severe Omicron disease.

## Methods and materials

### Data collection

The retrospective multicenter cohort of the SARS-CoV-2 ancestral strain has been described in previous studies [20]. The cohort of severe patients with Omicorn in this study was derived from 5 designated COVID-19 hospitals in Jiangsu Province, China. Retrospective data were approved by the Ethics Committee of Zhongda Hospital (Approval No. 2023ZDKYSB003). Between November 16, 2022, and January 30, 2023, adult COVID-19 patients admitted to the ICUs of participating hospitals were included in this study if they met the following inclusion criteria: (1) aged > 18 years; (2) laboratory-confirmed COVID-19; (3) severe respiratory failure requiring advanced respiratory support, circulatory shock, or multiple organ failure. There were no exclusion criteria.

### Clinical sample collection and ethical approval

Clinical peripheral blood and bronchoalveolar lavage fluid (BALF) samples used in this study were obtained from the Department of Critical Care Medicine, Zhongda Hospital, Southeast University, and approved by the Ethics Committee of Zhongda Hospital (Approval No. 2023ZDKYSB003). Paraffin-embedded tissue samples from COVID-19 patients and healthy controls (adjacent tissues of pulmonary hamartoma) were provided by lung transplant patients at Wuxi People’s Hospital Affiliated to Nanjing Medical University (Approval No. KY24001). Informed consent was obtained from all participants, and the study was approved by the institutional ethics committees.

### Cell culture and preparation

All cell line was purchased directly from Wuhan Procell Life Science & Technology Co., Ltd. According to the provider’s certification, the cell line was authenticated by STR profiling and tested negative for mycoplasma contamination. Upon receipt, we conducted our own mycoplasma testing using a PCR-based method and confirmed the absence of contamination. Cells were used for experiments at low passage numbers.

Cells were cultured under standard conditions. 293 T cells were maintained in DMEM complete medium (Gibco), while Jurkat cells and primary human peripheral blood CD4⁺ T cells were cultured in RPMI-1640 complete medium (Gibco). For CD4⁺ T cells, magnetic sorting (Miltenyi) was performed, followed by flow cytometry to confirm a CD3⁺/CD4⁺ double-positive rate >90%. Cells were stimulated with CD3/CD28 antibody-conjugated magnetic beads (Genscript) for 48 h before subsequent experiments.

### SARS-CoV-2 propagation, titration, and biosafety procedures

The viral variants used in this study included: a. The ancestral strain of SARS-CoV-2 (Wild Type, WT), using the international standard strain Wuhan-Hu-1 (RefSeq accession: NC_045512.2); b. The Omicron variant, subtype XBB.1.19, isolated from pharyngeal swab samples of locally confirmed cases in Jiangsu Province, China in 2023 and verified by next-generation sequencing to carry characteristic mutations in the spike protein of Omicron. In April 2023, XBB.1.19 was one of the predominant XBB sublineages circulating across multiple provinces in China (including Beijing) [21], and the isolate used in this study was derived from a local Jiangsu case to ensure regional representativeness. Furthermore, although differences in replacement dynamics among XBB sublineages are minimal, XBB.1 sublineage exhibits notably strong immune evasion, making it a lineage of particular biological interest.

All SARS-CoV-2 variants were passaged and amplified in Vero E6 cells, and viral titers were determined by the Plaque Assay. In vitro infection experiments were conducted in a Biosafety Level 3 (BSL-3) laboratory at the Jiangsu Provincial Center for Disease Control and Prevention. All laboratory personnel held the Highly Pathogenic Microorganism Laboratory Qualification Certificate issued by the National Health Commission of China. Infected samples were inactivated by water bath at 56°C for 30 minutes [22]; after inactivation, samples were transported to a BSL-2 laboratory for subsequent experiments.

### SARS-CoV-2 spike protein attachment to target cells

Purified SARS-CoV-2 Spike proteins (Genscript, Cat. No.: Z03483/Z03729) were commissioned to MedChemExpress (MCE) for Alexa Fluor 488 labeling. The final protein concentration was adjusted to 10 μM and stored in PBS. Test cells were digested, resuspended in pre-chilled PBS, and adjusted to a density of 1–2 × 10⁶ cells/mL. For each reaction, 100 μL of cell suspension was centrifuged, then mixed with 2 μL of labeled Spike protein (final concentration 200 nM) in a 1.5-mL Eppendorf tube. The mixture was gently vortexed and incubated for 30 min at room temperature in the dark, with 5-second vortexing every 10 min to maintain binding kinetic equilibrium. Non-specifically bound proteins were washed twice with PBS, and cells were transferred to flow cytometry tubes for analysis.

### Molecular docking simulation

The structural model of CD63 was obtained from the AlphaFold database. The trimeric structure of the HIV-1 gp41 membrane-proximal external region (MPER), along with its transmembrane domain and cytoplasmic tail, was retrieved from the Protein Data Bank (PDB ID: 7LOI, residue 660-856). The receptor-binding domain (RBD) of the SARS-CoV-2 spike protein, in both the wild-type (WT) and Omicron variant, was obtained from the Protein Data Bank (6M0J and 7T9O), respectively. Molecular docking simulations were performed using the HADDOCK software package with default parameters [23].

### Pseudovirus infection assay

Twenty-four hours prior to the experiment, 293 T or Jurkat cells were adjusted to a density of 1 × 10⁴ viable cells per well and seeded into 96-well black clear-bottom culture plates (Beyotime). When cell confluency reached approximately 30%, COVID-19 Spike Protein Pseudovirus (Yeasen, Cat. No. 11906ES/11991ES) was added to the plates at a pre-determined multiplicity of infection (MOI). After 6 h, the virus-containing supernatant was removed, and cells were replenished with pre-warmed complete medium (containing 2% FBS) for continued culture over 48 h. Fluorescent intensity was monitored periodically during this period.

### Transmission electron microscopy (TEM)

Sample preparation was performed by Servicebio Technology Co., Ltd. (Wuhan, China) following standard protocols. Images were acquired using a transmission electron microscope at an accelerating voltage of 80 kV, with magnification ranging from 2500× to 30,000×.

### Construction of a biomimetic lung microphysiological system

The system was installed according to standard methods [24]. Calu-3 epithelial cells were transfected with SARS-CoV-2 Spike protein, and 24 h later, the cells were digested, resuspended, and seeded into the upper chamber of the biomimetic lung microphysiological system. The device was cultured overnight at standard conditions to allow epithelial cell adhesion. Following adhesion, the medium in the upper chamber was aspirated, and T cells (1 × 10⁵ cells/mL) was perfused from the lower chamber at a flow rate of 1 µL/min to establish an air-liquid culture interface. Apoptosis was detected 24 h later.

### Flow cytometric analysis

For clinical samples, peripheral blood mononuclear cells (PBMCs) were isolated from peripheral blood using SepMate™ centrifugation tubes (STEMCELL). Cell viability was stained with Zombie NIR™ Fixable Viability Kit (Biolegend, 423105). Membrane proteins CD45 (eBioscience, 368508) and CD3 (eBioscience, 69-0038-42) were labeled at room temperature. Following fixation and permeabilization (eBioscience protocol), cells were stained for SARS-CoV-2 nucleocapsid protein using an antibody (Abcam, ab302552).

Cultured cells were resuspended in 1× Annexin V binding buffer (containing 10 mM HEPES, 140 mM NaCl, 2.5 mM CaCl₂, pH 7.4) at a density of 1 × 10⁶ cells/mL. For each sample, 100 μL of cell suspension was mixed with 2 μL of APC-Annexin V (BioLegend) and 1 μL of Propidium Iodide (BioLegend), gently vortexed, and incubated for 15 min at room temperature in the dark. After two washing steps, cells were analyzed by flow cytometry.

### Real-time PCR

Total RNA was extracted via TRIzol reagent (TaKaRa). cDNA synthesis was performed via HiScript III Reverse Transcriptase (Vazyme). Real-time PCR was performed using AceQ Universal SYBR qPCR Master Mix (Vazyme).

**Table:** 

Gene	sequence (5’-3’)
**Human**	
GAPDH-F	5’-GGAGCGAGATCCCTCCAAAAT-3'
GAPDH-R	5’-GGCTGTTGTCATACTTCTCATGG-3'
**SARS-CoV-2**	
ORF1ab-F	5’-CCCTGTGGGTTTTACACTTAA-3'
ORF1ab-R	5’-TCAGCTGATGCACAATCGT-3'
N-F	5’-GGGGAACTTCTCCTGCTAGAAT-3'
N-R	5’-CAGCTTGAGAGCAAAATGTCTG-3'

### Western blot

Western blot was performed following standard protocols. The primary antibodies used were: CD3 (Proteintech, 60181-1-lg), CD63 (Abcam, ab315108), DYKDDDDK-Tag (Cell Signaling Technology, 14793), His-Tag (Cell Signaling Technology, 12698), SARS-CoV-2 Nucleocapsid Protein (Cell Signaling Technology, 26369), p-MLKL (Abcam, ab187091), MLKL (Abcam, ab184718), Cleaved Caspase-3 (Cell Signaling Technology, 9664T), and BCL2L13 (Abcam, ab203516).

### Cell death-associated gene sets

Apoptosis-related genes (161 genes, MSigDB database [25], https://www.gsea-msigdb.org/gsea/msigdb, GOBP_NECROPTOTIC_PROCESS gene set): ADD1, AIFM3, ANKH, ANXA1, APP, ATF3, AVPR1A, BAX, BCAP31, BCL10, BCL2L1, BCL2L10, BCL2L11, BCL2L2, BGN, BID, BIK, BIRC3, BMF, BMP2, BNIP3L, BRCA1, BTG2, BTG3, CASP1, CASP2, CASP3, CASP4, CASP6, CASP7, CASP8, CASP9, CAV1, CCNA1, CCND1, CCND2, CD14, CD2, CD38, CD44, CD69, CDC25B, CDK2, CDKN1A, CDKN1B, CFLAR, CLU, CREBBP, CTH, CTNNB1, CYLD, DAP, DAP3, DCN, DDIT3, DFFA, DIABLO, DNAJA1, DNAJC3, DNM1L, DPYD, EBP, EGR3, EMP1, ENO2, ERBB2, ERBB3, EREG, ETF1, F2, F2R, FAS, FASLG, FDXR, FEZ1, GADD45A, GADD45B, GCH1, GNA15, GPX1, GPX3, GPX4, GSN, GSR, GSTM1, GUCY2D, H1-0, HGF, HMGB2, HMOX1, HSPB1, IER3, IFITM3, IFNB1, IFNGR1, IGF2R, IGFBP6, IL18, IL1A, IL1B, IL6, IRF1, ISG20, JUN, KRT18, LEF1, LGALS3, LMNA, PLPPR4, LUM, MADD, MCL1, MGMT, MMP2, NEDD9, NEFH, PAK1, PDCD4, PDGFRB, PEA15, PLAT, PLCB2, PMAIP1, PPP2R5B, PPP3R1, PPT1, PRF1, PSEN1, PSEN2, PTK2, RARA, RELA, RETSAT, RHOB, RHOT2, RNASEL, ROCK1, SAT1, SATB1, SC5D, SLC20A1, SMAD7, SOD1, SOD2, SPTAN1, SQSTM1, TAP1, TGFB2, TGFBR3, TIMP1, TIMP2, TIMP3, TNF, TNFRSF12A, TNFSF10, TOP2A, TSPO, TXNIP, VDAC2, WEE1, XIAP.

Pyroptosis-related genes [26] (52 genes): BAK1, BAX, CASP1, CASP3, CASP4, CASP5, CHMP2A, CHMP2B, CHMP3, CHMP4A, CHMP4B, CHMP4C, CHMP6, CHMP7, CYCS, ELANE, GSDMD, GSDME, IL18, IL1A, IL1B, IRF1, IRF2, TP53, TP63, AIM2, CASP6, CASP8, CASP9, GPX4, GSDMA, GSDMB, GSDMC, IL6, GZMB, HMGB1, NLRP6, NLRP7, NOD1, NOD2, PJVK, PLCG1, PRKACA, PYCARD, SCAF11, TIRAP, TNF, GZMA, NLRC4, NLRP1, NLRP2, NLRP3.

Necroptosis-related genes (49 genes, MSigDB database [25], https://www.gsea-msigdb.org/gsea/msigdb, GOBP_NECROPTOTIC_PROCESS gene set): DNM1L, PPIF, RBCK1, RIPK3, PARP1, CYLD, NLRP6, PGAM5, MLKL, IPMK, SLC25A4, BIRC2, BIRC3, FAS, FASLG, ITPK1, MIR101-1, MIR103A1, MIR107, MIR214, MIR22, MIR221, MIR223, MUTYH, PRKN, TRPM7, ADPRS, RNF31, PELI1, MIR485, PYGL, BOK, TLR3, TNF, TP53, ZBP1, FZD9, ITCH, CASP6, CASP8, OGT, YBX3, CAV1, RIPK1, FADD, CFLAR, AIFM1, ARHGEF2, SPATA2.

### Proteomics and differential analysis

Differential proteins between Omicron and WT bystander T cells were identified with limma (two-group design); proteins with *p* ≤ 0.05 were ranked by log2 fold-change (Omicron vs WT), and the top ten upregulated candidates were prioritized for functional screening (listed in Supplementary Table S1).

### Statistical analysis

The sample size for each group (*n* = 3–5) was chosen based on common practices in the field for analogous experiments [10, 27] and our preliminary data, which indicated that this size reliably estimates the mean and variance of our primary outcomes. Upon collection, samples were randomly allocated to experimental groups using a sequence generated by the =RAND() function in Microsoft Excel. All subsequent processing and analyses were performed in a randomized order to minimize batch effects and bias.

Experimental data are presented as mean ± standard deviation (±SD). All statistical analyses were performed using GraphPad Prism 9.5 and R 4.3.1 statistical computing software. The following comparison strategies were employed: Two-group comparisons: Two-tailed unpaired Student’s *t* test was used when data followed a normal distribution (equal variance confirmed by F-test). Multi-group comparisons: One-way analysis of variance (ANOVA) followed by Tukey’s post-hoc test for multiple comparisons. Non-parametric tests: Mann-Whitney *U* test (two groups) or Kruskal-Wallis test (multiple groups) were applied when data did not meet the normality assumption. Data were considered statistically significant when the *P* value was <0.05.

To address baseline imbalances between the ancestral-strain and Omicron ICU cohorts, we conducted additional multivariable analyses and propensity score weighting. Peripheral lymphocyte counts were log-transformed using the natural logarithm (ln) for regression modeling. We fitted an ordinary least squares linear regression with variant (Omicron vs. ancestral) as the key predictor, adjusting for age (restricted cubic splines; 4 knots), sex, and comorbidities (hypertension, diabetes, chronic kidney disease, coronary artery disease, malignancies, COPD, cirrhosis). HC3 heteroskedasticity-robust standard errors were used for inference. We also calculated propensity scores for Omicron infection using the same covariates and applied overlap weighting to estimate the average treatment effect (ATE) on lymphocyte count. In addition, we performed quantile regression to compare lymphocyte distributions between groups at the median (*τ* = 0.5) and lower decile (*τ* = 0.1), with covariate adjustment as above; 95% confidence intervals for the 10th-percentile effect were obtained via bootstrap resampling (1000 iterations). Finally, a multivariable logistic regression model assessed the odds ratio (OR) of severe lymphopenia (defined as absolute lymphocyte count <1.0 × 10^9/L) in Omicron vs. ancestral patients. To visualize distributional differences, we generated overlap-weighted empirical cumulative distribution functions (ECDFs) and a quantile treatment effect (QTE) curve illustrating the relative lymphocyte count in Omicron compared to ancestral across the lymphocyte quantile range.

## Results

### Profound lymphopenia highlights omicron’s immune dysregulation

We performed a retrospective analysis of ICU cases from 19 designated COVID-19 hospitals in China during the prevalence of the ancestral SARS-CoV-2 strain (January 1, 2020, to February 29, 2020; 733 cases) and 5 designated COVID-19 hospitals in China during the Omicron prevalence period (November 16, 2022, to January 30, 2023; 678 cases). Comparative results showed that the cohort of critically ill Omicron-infected patients had a higher proportion of males (*p* = 0.0077), an older age (*p* < 0.0001), a higher rate of comorbidities, and higher Apache II and SOFA scores (*p* < 0.0001). Of particular note, lymphocyte counts in the Omicron group were lower than those in severe patients infected with the ancestral strain (Table 1). This suggests that Omicron may mediate unique mechanisms of immune injury; despite evolving to primarily cause upper respiratory tract symptoms, the mortality rate among Omicron-infected individuals progressing to ICU admission is not lower than that of earlier strains. After adjusting for the differences in age and comorbidities, the variant-associated disparity in lymphocyte counts was no longer statistically significant. In the adjusted log-linear model, the Omicron group’s geometric mean lymphocyte count was 0.962 times that of the ancestral group (95% CI 0.872–1.060; *p* = 0.4325). With propensity-score overlap weighting, the geometric-mean ratio was 0.963 with 95% CI 0.898–1.033 and *p* = 0.2950.

**Table 1:** Comparative characteristics of severe COVID-19 patients across SARS-CoV-2 variants.

	WT severe (*n* = 733)	Omicron severe (*n* = 678)	*p* value
**Demographics**			
Sex (females)^a^	256 (34.92)	192 (28.32)	0.0077^c^
Age (years)^b^	65 (56, 73)	79 (69, 86)	<0.0001^d^
BMI^b^	23.95 (22.04, 26.60)	23.44 (20.24, 25.49)	0.0041^d^
**Comorbidities**			
Hypertension^a^	308 (42.02)	415 (61.21)	<0.0001^c^
Diabetes^a^	138 (18.83)	215 (31.71)	<0.0001^c^
Chronic kidney disease^a^	13 (1.77)	47 (6.93)	<0.0001^c^
Coronary artery disease^a^	93 (12.69)	148 (21.83)	<0.0001^c^
Malignancies^a^	24 (3.27)	70 (10.32)	<0.0001^c^
COPD^a^	37 (5.05)	56 (8.26)	0.0151^b^
Cirrhosis^a^	11 (1.50)	5 (0.74)	0.1761^b^
**Severity Score of illness**			
Apache II^b^	10 (7, 14)	20.5 (15, 26)	<0.0001^d^
SOFA score^b^	4 (1, 5)	8 (6, 11)	<0.0001^d^
**Vital Signs**			
Body temperature(°C)^b^	36.8 (36.5,37.6)	37 (36.5, 37.8)	<0.0001^d^
Respiratory Rate (bpm)^b^	23 (20, 29)	22 (18, 29)	0.0001^d^
Heart rate (bpm)^b^	92 (81, 104)	95 (83, 111)	0.1094^d^
**Clinical Laboratory Results**			
Blood pH^b^	7.44 (7.40, 7.48)	7.41 (7.35, 7.46)	<0.0001^d^
Oxygen Saturation (SpO₂%)^b^	93 (86, 97)	94 (89, 97)	0.1388^d^
PaO2^b^	61.8 (40.45, 83.25)	89.55 (68.15, 127.5)	<0.0001^d^
PaCO2^b^	38 (31.85, 60)	35.9 (29.85, 45.6)	0.0071^d^
PaO2/FiO2(mmHg)^b^	115.33 (67.42, 175.60)	238.11 (156.27, 317.15)	<0.0001^d^
Lymphocyte (10⁹/L)^b^	0.64 (0.43, 0.96)	0.57 (0.38, 0.82)	0.0071^d^
**Respiratory support**			
Oxygen therapy^a^	149 (20.33)	306 (45.13)	<0.0001^c^
HFNC^a^	320 (43.66)	105 (15.49)	0.0019^c^
Mechanical ventilation^a^	229 (31.24)	267 (39.38)	0.0014^c^
ECMO^a^	35 (4.77)	5 (0.74)	<0.0001^c^
**LOS in ICU (days)**^**b**^	12 (6, 25)	11 (5, 20)	0.1503^d^
**LOS in hospital (days)**^**b**^	15 (9, 24)	13 (7, 22)	0.0111^d^
**ICU mortality**^a^	475 (64.80)	474 (69.91)	0.0410^c^
**28-day mortality**^a^	394 (53.75)	360 (53.09)	0.8055^c^

*HFNC* high-flow nasal cannula oxygen therapy, *LOS* the length of stay.

^a^– Frequency (%).

^b^– Median (IQR).

^c^– Chi-square test (Statistical tests used).

^d^– Mann-Whiney U test (Statistical tests used).

Recognizing that mean differences can obscure heterogeneity, we examined effects at the lower tail of lymphocyte distribution (*τ* = 0.10). The estimate suggested approximately an 11% reduction in lymphocyte counts in Omicron cases at this quantile (coef ~−0.114; bootstrap *p* = 0.257). Although this trend did not reach statistical significance, it is consistent with the mechanistic hypothesis that Omicron may preferentially affect extreme lymphocyte depletion. The overlap-weighted ECDF curves (Supplementary Fig. 1A) illustrate that the Omicron curve lies above the ancestral curve in the lymphocyte-low range, reflecting an increased cumulative probability of severe lymphopenia in the Omicron group. Correspondingly, the QTE curve remained below 1.0 at the lower quantiles (Supplementary Fig. 1B), indicating a potential excess of extreme T-cell depletion in Omicron cases even though the central tendency of lymphocyte counts was similar between groups.

### Omicron exhibits conserved T cell infectivity

**Fig. 1:**
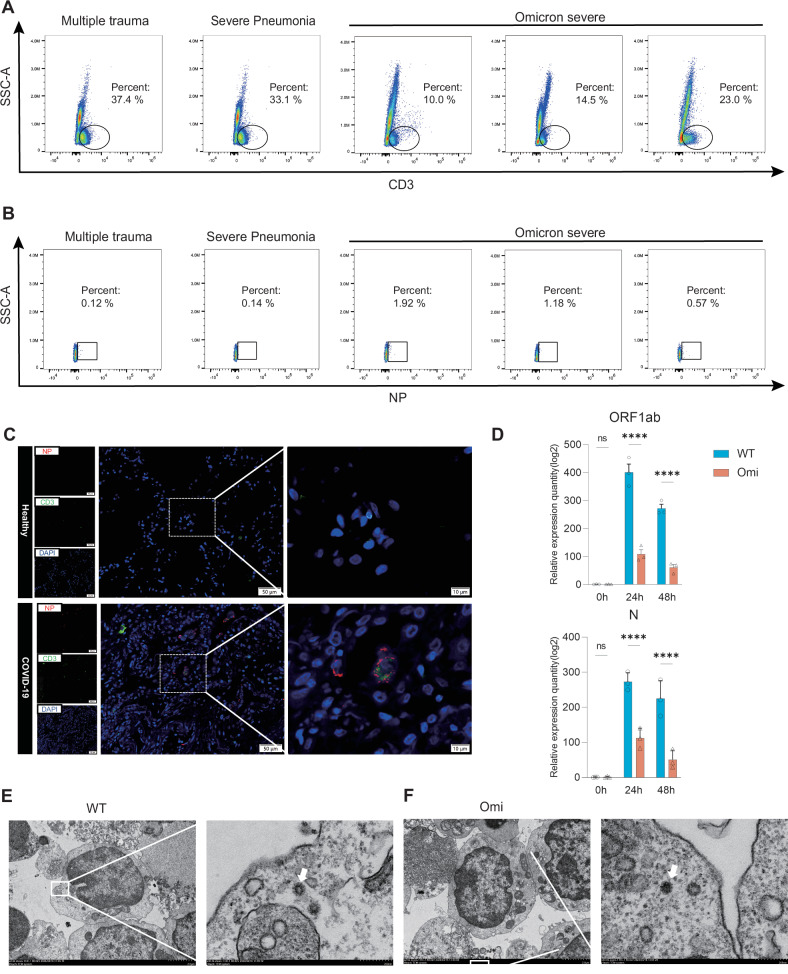
Omicron exhibit conserved T cell infectivity. **A**, **B** Flow cytometry analysis of peripheral blood CD3 + T lymphocyte proportions and SARS-CoV-2-specific NP-positive rates in CD3 + T cells from multiple trauma patients, severe pneumonia patients, and severe COVID-19 patients. **C** Immunofluorescence staining of lung tissue sections shows co-localization of SARS-CoV-2 NP protein (red) and CD3 + T cells (green). Scale bars = 50 μm/10 μm. **D** RT-qPCR detection of SARS-CoV-2 signature gene fragments N and ORF1ab in Jurkat cells infected with ancestral strain and Omicron at 24 h and 48 h. **E**, **F** Transmission electron microscopy of primary human peripheral blood CD4 + T lymphocytes infected with ancestral strain and Omicron for 24 h. White arrows indicate intracellular coronavirus particles. Scale bars = 2 μm/200 nm.

Lymphocyte count reduction in COVID-19 patients is primarily attributed to T cell alterations [2]. We demonstrated that CD3 + T cell counts in severe Omicron-infected patients were lower than those in severe non-infected patients (multiple trauma) and severe pneumonia patients (Fig. 1A). Previous studies have reported a high proportion of viremia in ancestral strain COVID-19 patients [28, 29], indicating viral positivity in peripheral blood. Consistently, NP protein was detected in peripheral CD3⁺ T cells of severe Omicron cases (Fig. 1B). To verify T cell infectivity by Omicron, we used lung tissue sections from severe Omicron patients, Jurkat cells, and primary T cells. Compared with controls, CD3⁺ T cells in severe Omicron patient lungs co-localized with SARS-CoV-2 Nucleocapsid Protein (NP) (Fig. 1C). In vitro infection of Jurkat cells with ancestral and Omicron variants showed both variants directly infected T cells, with significantly higher viral loads for the ancestral strain than Omicron (Fig. 1D). This contrasts with Omicron’s evolutionary strategy in epithelial cells—while exhibiting stronger epithelial infectivity than the ancestral variant, Omicron shows reduced lymphocyte affinity. Peripheral blood primary CD4⁺ T lymphocytes were infected in vitro with both variants (MOI = 0.05), and transmission electron microscopy detected viral particles (white arrows) within cells (Fig. 1E). Collectively, these data indicate conserved T cell infectivity across SARS-CoV-2 variants.

### CD63 facilitates SARS-CoV-2 Infection of T cells via ACE2-independent pathway

**Fig. 2:**
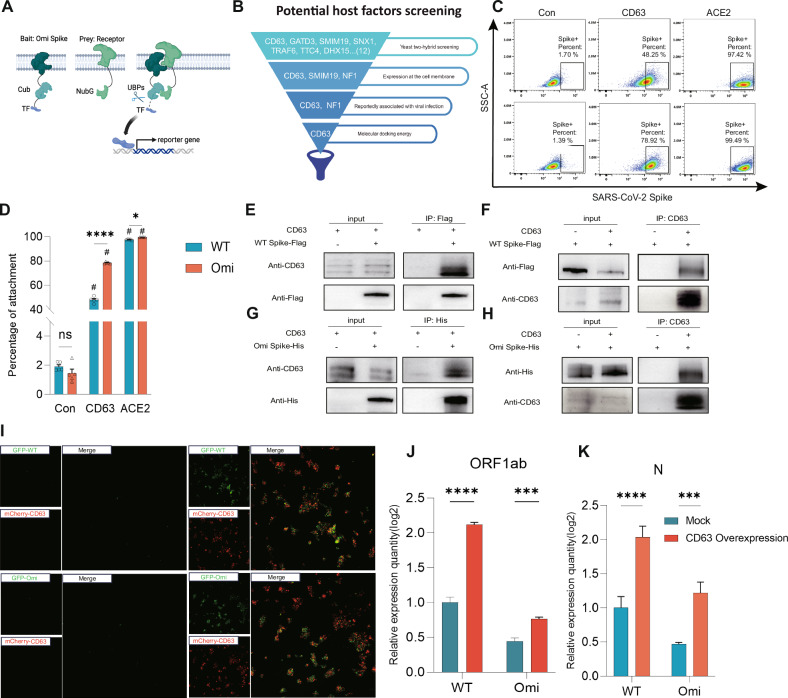
CD63 facilitates SARS-CoV-2 infection of T cells via an ACE2-independent pathway. **A** Schematic of yeast two-hybrid assay: Bait protein (Omicron Spike) fused with ubiquitin Cub to BD vector, prey protein fused with ubiquitin NubG to AD vector. Upon protein interaction in the yeast strain, intact ubiquitin is recognized and cleaved by UBPs, allowing transcription factors to activate reporter genes. **B** Schematic of candidate receptor protein screening. **C**, **D** Flow cytometry analysis of CD63-mediated adsorption efficiency for different variants: Alexa Fluor 488-Spike was incubated with CD63-overexpressing/control cells at room temperature for 30 min, washed twice, and Alexa Fluor 488-positive cell proportion was detected. **E**, **F**, **G**, **H** Co-IP analysis of interactions between ancestral/Omicron Spike and CD63. **I** Immunofluorescence assay of CD63-overexpressing 293 T cells promoting pseudovirus infection. **J**, **K** RT-qPCR detection of SARS-CoV-2 ORF1ab gene expression in Jurkat and Jurkat_CD63 cells infected with ancestral/Omicron strains (MOI = 0.05) for 24 h.

It has been established that T cells can be directly infected by distinct SARS-CoV-2 variants, despite the extremely low expression of ACE2 in T cell subsets [30]. This observation implies that SARS-CoV-2 may employ an ACE2-independent pathway to infect T cells. In this study, a yeast two-hybrid membrane system was constructed (Fig. 2A), through which 12 candidate proteins interacting with the Spike protein were co-screened. Given the potential for non-physiological interactions detected by the yeast two-hybrid system, further screening of these candidate proteins was performed (Fig. 2B). Proteins expressed on the human cell membrane included CD63, SMIM19, and NF1. A literature review revealed that CD63 and NF1 have been previously associated with HIV viral infection [12, 31]. Molecular docking analyses of CD63 and NF1 with the SARS-CoV-2 Spike structure showed that CD63 exhibited stronger binding affinity (Supplementary Fig. 2D). Consequently, CD63 was selected as a potential receptor for SARS-CoV-2 for further validation. Affinity analysis showed that CD63 has a higher binding affinity with the Omicron variant than WT, but lower binding affinity with gp41 (Supplementary Fig. 2E), Daniel Ivanusic et al. reported that CD63 interacts with the gp41 protein of HIV through its large extracellular loop (LEL) [32]. Our findings suggest that SARS-CoV-2 may also utilize the RBD of its spike protein to interact with the LEL of CD63. This difference between the two variants aligns with viral evolutionary logic, where enhanced cellular affinity is a key adaptive trait, consistent with the increased ACE2 binding affinity observed in the Omicron Spike protein.

**Fig. 3:**
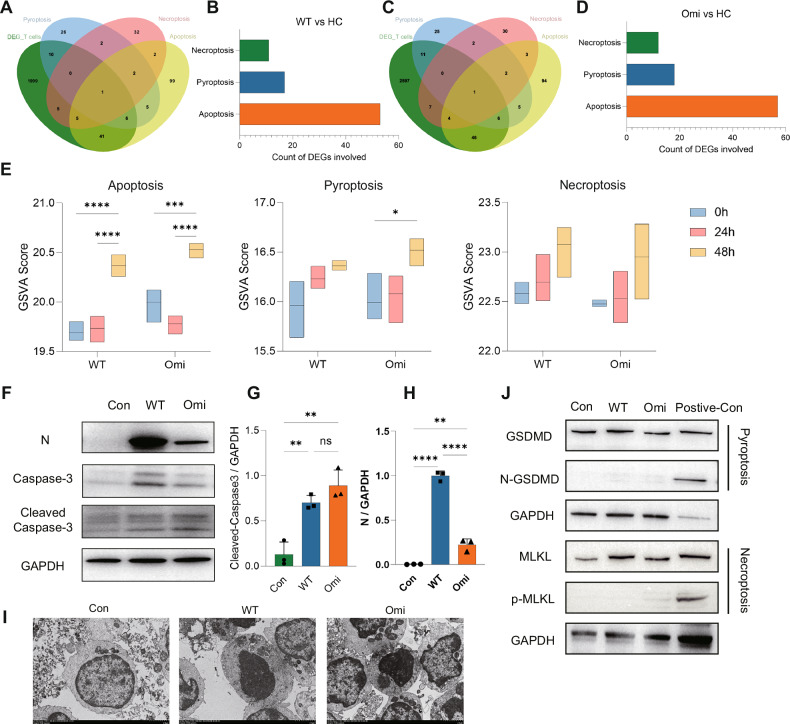
Omicron and ancestral SARS-CoV-2 conservedly induce late-stage apoptosis in T lymphocytes. **A**, **B** Intersection analysis of differentially expressed genes (DEGs) between ancestral SARS-CoV-2-infected individuals and healthy with cell death-related gene sets, showing the highest number of apoptosis-related genes. **C**, **D** Intersection analysis of DEGs between Omicron-infected individuals and healthy with cell death-related gene sets, with the highest number of apoptosis-related genes. **E** GSVA pathway enrichment analysis of transcriptome data to determine the primary mode of T cell death. **F** Western blot validation of T cell apoptosis pathway activation. **G** Densitometry analysis of Cleaved Caspase-3. **H** Densitometry analysis of N protein. **I** Transmission electron microscopy to confirm the primary mode of cell death. **J** Western blot excluding other pathways of cell death.

To investigate whether CD63 mediates viral adsorption, Alexa Fluor 488-conjugated Spike proteins were designed and incubated with 293 T cells (negative control), 293T-ACE2 cells (positive control overexpressing ACE2), and 293T-CD63 cells (Supplementary Fig. 2A, B). Flow cytometry analyses showed significantly higher Spike adsorption efficiency in 293T-CD63 cells compared to the negative control group, with the Omicron variant exhibiting a higher binding ratio, consistent with the aforementioned molecular docking results (Fig. 3C, D), indicating that the membrane protein CD63 facilitates Spike binding. Co-immunoprecipitation assays further confirmed protein-protein interactions between CD63 and Spike proteins from both the wild-type strain (WT Spike-Flag) and the Omicron variant (Omi Spike-His) (Fig. 2E–H). Additionally, overexpressing CD63 exhibited significantly enhanced entry of SARS-CoV-2 pseudoviruses coated with Spike proteins from different variants (Fig. 2I, Supplementary Videos). To validate these findings in physiologically relevant T cells, Jurkat cell lines stably overexpressing CD63 were generated (Supplementary Fig. 2C). Infection with both the ancestral SARS-CoV-2 strain and the Omicron variant demonstrated that CD63 overexpression significantly increased the expression levels of viral markers N and ORF1ab compared to control (Fig. 2J, K).

Previous studies have reported that CD63 overexpression combined with low pH promotes syncytia formation and increases Lujo virus infectivity [15]. Additionally, low pH has been associated with SARS-CoV-2 infection and COVID-19 severity in infected patients [33]. Based on this, we hypothesized that low pH may enhance SARS-CoV-2 infectivity. Control and CD63-overexpressing cells were treated with low pH (incubation in pH 4.0 PBS for 5 min), followed by replacement with normal medium and incubation at 37°C for 30 min prior to microscopy or pseudovirus infection. Results showed that CD63 overexpression increased cell fusion, a phenomenon further accentuated by low-pH treatment (Supplementary Fig. 2G). Given that increased syncytia may facilitate viral entry, we infected CD63-overexpressing cells with or without low pH pretreatment using ancestral SARS-CoV-2 and Omicron pseudoviruses. Low pH pretreatment alone did not increase viral load, but in CD63-overexpressing cells, low pH effectively promoted viral entry (Supplementary Fig. 2H, I), potentially linked to enhanced cell fusion, syncytia formation, and endocytic efficiency. To explore the endocytic pathway underlying CD63-mediated infection, we used Dynasor (inhibitor of clathrin-mediated endocytosis), Nystatin (inhibitor of caveolin-mediated endocytosis), and Blebbistatin (inhibitor of macropinocytosis) in cells with low pH treatment combined with CD63 overexpression. Results indicated that CD63-mediated SARS-CoV-2 infection depends on clathrin-mediated endocytosis (Supplementary Fig. 2J, K).

Collectively, these results demonstrate that CD63 mediates SARS-CoV-2 infection of T cells by directly binding to the viral Spike protein, independent of the classical ACE2-TMPRSS2 pathway. This mechanism is highly conserved across different SARS-CoV-2 variants.

### Omicron and ancestral SARS-CoV-2 conservedly induce late-stage apoptosis in T lymphocytes

Programmed cell death of host cells following direct pathogen infection serves as a self-defense mechanism. To investigate the specific mode of cell death mediated by SARS-CoV-2 in T lymphocytes, we collected gene sets related to apoptosis, pyroptosis, and necroptosis and analyzed the activity of cell death pathways in both in vivo and in vitro SARS-CoV-2-infected samples. Single-cell RNA-seq data analysis of bronchoalveolar lavage fluid (BALF) from COVID-19 patients revealed that differentially expressed genes (DEGs) in T cells between COVID-19 patients and healthy volunteers overlapped with multiple cell death-related gene sets (Fig. 3A, C). Among these, apoptosis-related genes exhibited the highest number and proportion of intersections with DEGs, consistently observed in both ancestral strain and Omicron variant infections (Fig. 3B, D, Supplementary 3A, B). Notably, Caspase-8—a molecular switch for apoptosis, pyroptosis, and necroptosis—was identified as a common intersecting gene across multiple cell death pathways [34]. This suggests that COVID-19 activates multiple cell death pathways, with apoptosis being the most prominent, and this mechanism is conserved across SARS-CoV-2 variants.

**Fig. 4:**
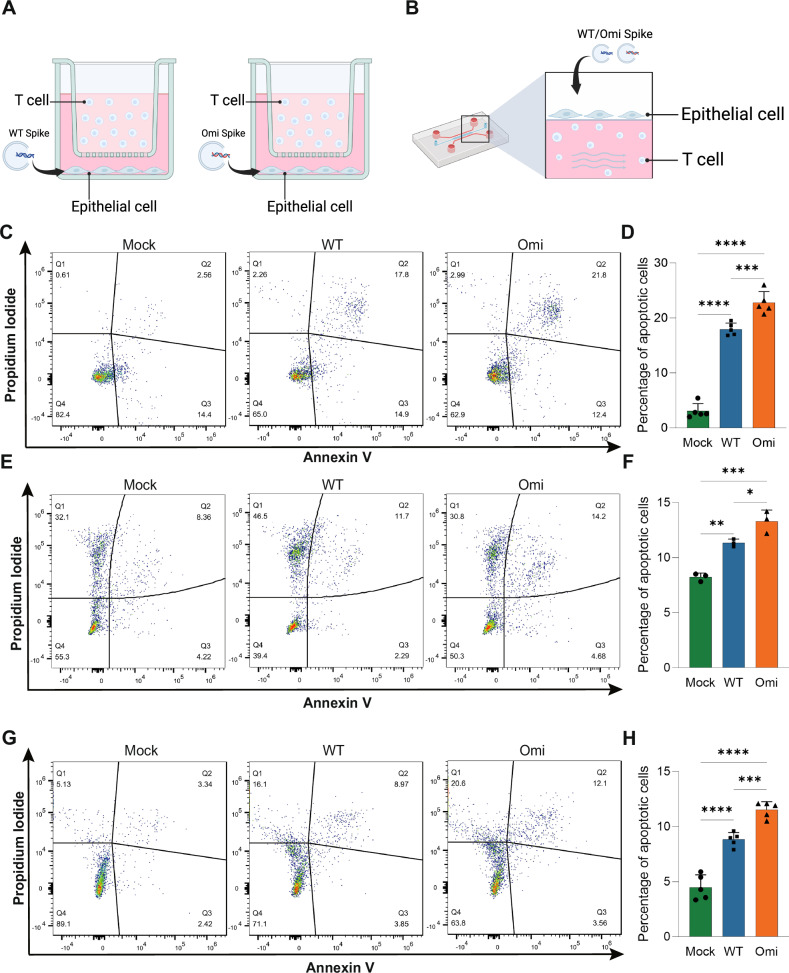
Enhanced bystander T cell apoptosis induced by Omicron via epithelial-immune crosstalk. **A** Schematic of the epithelial-T cell co-culture system: Calu-3 epithelial cells were transfected with spike to mimic infection, and suspension T cells (Jurkat or primary human T cells) were added for co-culture after 24 h. **B** Schematic of the Biomimetic Lung Microphysiological System: Calu-3 cells were seeded in the upper chamber, and Jurkat cells were inoculated in the flowing medium of the lower chamber to mimic the alveolar air-liquid interface. **C**, **D** Flow cytometry analysis of apoptosis in co-cultured Jurkat cells stained with Propidium Iodide and Annexin V. **E**, **F** Flow cytometry analysis of apoptosis in co-cultured primary T cells stained with Propidium Iodide and Annexin V. **G**, **H** Flow cytometry analysis of apoptosis in Jurkat cells flowing through the lower chamber of the Biomimetic Lung Microphysiological System, stained with Propidium Iodide and Annexin V.

Further analysis of RNA-seq data from in vitro-infected Jurkat cells (Supplementary 4A) used Gene Set Variation Analysis (GSVA) to calculate enrichment scores for cell death-related genes. Results showed that infection with two SARS-CoV-2 variants primarily induced apoptosis in T cells. Statistically significant differences in apoptosis scores were observed between infected groups at 48 h versus 24 h/controls post-infection (Fig. 4E), indicating that apoptosis occurs in the late stage of infection. Western blot analysis of Jurkat T cell infection samples confirmed upregulation of the apoptosis marker Cleaved Caspase-3 in infected groups, with no significant differences in induction levels between variants (Fig. 4F, G). Higher intracellular viral loads were observed in the ancestral SARS-CoV-2 strain-infected group (Fig. 4F, H), consistent with the above RT-qPCR results. Transmission electron microscopy of primary human peripheral blood T cells infected in vitro revealed typical apoptotic features, including cell shrinkage, cytoplasmic condensation, and nuclear pyknosis (Fig. 4I). No significant upregulation was detected for N-Gasdermin D or p-MLKL (Fig. 4J), although pyroptosis gene sets showed upregulation in Omicron-infected cells at 48 h compared to controls. Collectively, both the Omicron variant and the ancestral strain primarily induce T cell apoptosis upon direct infection, with apoptosis occurring in the late stage of infection.

In addition, multi-omics data (RNA-seq and proteomic) also confirmed that molecular regulatory networks induced by the two variants during infection were highly time-dependent rather than variant-specific, as evidenced by significantly separated data profiles at different infection time points (0/24/48 h) and highly overlapped molecular features across variants at the same time point (Supplementary 4B, C, D, E, F). These findings suggest that although Omicron exhibits stronger immune evasion capacity in clinical phenotypes, its molecular reprogramming mechanisms upon direct T lymphocyte infection are inherited from the conserved pathways of the ancestral strain. Phenotypic differences may arise from structural variations in viral surface proteins (Spike) rather than differential remodeling of host intracellular regulatory networks. This discovery provides a new molecular perspective for understanding SARS-CoV-2 variant pathogenesis: viral components other than spike proteins maintain evolutionary conservation in hijacking core host functions.

### Enhanced bystander T cell apoptosis induced by Omicron via epithelial-immune crosstalk

The similar apoptosis-inducing capacity between the Omicron variant and the ancestral strain is insufficient to explain the lower T cell counts in severe Omicron patients [5]. We hypothesized that intercellular cross-talk might account for this discrepancy, proposing that infection of other cell types could mediate bystander T cell apoptosis. To explore this, we performed intercellular communication network analysis using scRNA-seq data and found dense signaling interactions between T cells and other immune cells, as well as epithelial cells (Epi) (Supplementary Fig. 5A–C). Given that epithelial cells are the primary target of SARS-CoV-2, we designed a co-culture system of epithelial cells and T cells (Fig. 4A). Epithelial Calu-3 cells were transfected with Spike plasmids of the ancestral strain or Omicron variant (Spike was selected as the infection-mimicking component due to its high mutation density in Omicron) to simulate viral infection. After 24 h, Jurkat/Primary T cells were co-cultured with the transfected epithelial cells in Transwell chambers, and apoptosis was assessed 24 h later. Flow cytometry showed significantly increased apoptosis in the simulated infection groups compared to the control, with a higher apoptosis proportion in the Omicron group than in the ancestral strain group (Fig. 4C–F).

To validate these findings, we used self-developed biomimetic lung microphysiological system to simulate the real air-liquid interface of the lung (Fig. 4B). This device has been previously validated for mimicking epithelial-immune cell interactions in prior studies [24]. Results were consistent with the Transwell co-culture system: infection of epithelial cells induced bystander T cell apoptosis, and Omicron elicited stronger bystander T cell apoptosis (Figs. 4G, 4H). These data suggest that the “epithelial-immune axis” in SARS-CoV-2 infection may represent an additional critical factor in T cell damage.

### Omicron potentiates bystander T cell apoptosis through the GDF15-BCL2L13 signaling axis

**Fig. 5:**
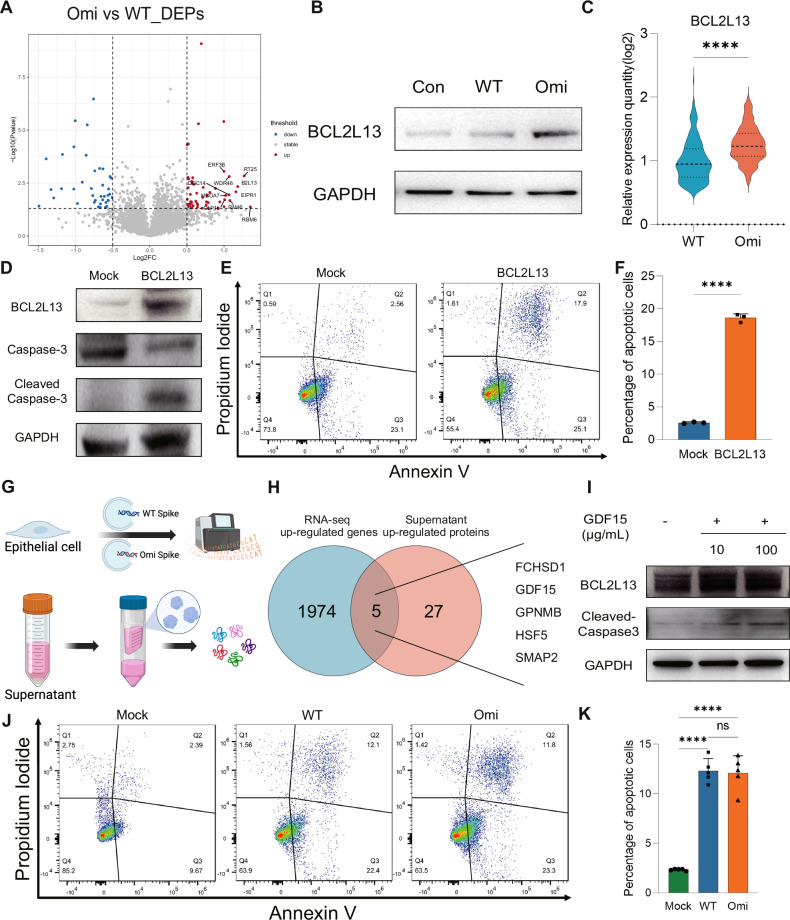
Omicron potentiates bystander T cell apoptosis through the GDF15-BCL2L13 signaling axis. **A** Volcano plot of differentially expressed proteins in bystander T cells from co-culture systems, comparing Omicron and ancestral strain groups. **B** BCL2L13 is specifically overexpressed in Omicron-induced bystander T cells. **C** Single-cell transcriptomic analysis of relative BCL2L13 expression in alveolar T cells, with significantly higher expression in Omicron than in the ancestral strain. **D** BCL2L13 overexpression induces activation of the T cell apoptosis pathway. **E**, **F** BCL2L13 overexpression increases the proportion of apoptotic T cells. **G** RNA-sequencing of epithelial cells and proteomic sequencing of cell supernatants from co-culture systems were performed to screen for extracellular inducers of high-intensity T cell apoptosis. **H** Intersection analysis of upregulated molecules from transcriptomic and proteomic data identified five candidate molecules: FCHSD1, GDF15, GPNMB, HSF5, and SMAP2. **I** Stimulation of Jurkat cells with the above candidate recombinant proteins showed that GDF-15 effectively induced activation of the T cell apoptosis pathway. **J**, **K** Knockdown of BCL2L13 in Jurkat cells abolished excessive Omicron-induced bystander T cell apoptosis.

Elucidating the mechanism by which Omicron induces stronger bystander T cell apoptosis is critical for understanding the immune exhaustion and prognostic differences between severe Omicron and the ancestral strain. In this study, proteomic analysis was performed on bystander T cells induced under simulated infection conditions with different variants. A total of 53 upregulated differentially expressed proteins(limma; *p* ≤ 0.05) were identified (Fig. 5A), the ten most upregulated proteins (ranked by log2 fold-change) taken forward for functional screening were RBM6, MRPS25 (RT25), BCL2L13, EIPR1, MRPL46 (RM46), WDR46, DGCR14 (DGC14), MBOAT7 (MBOA7), CLPTM1L (CLP1L), and GSPT2/eRF3b (ERF3B). Functional screening of the Top 10 proteins (Supplementary Table 1) revealed that only BCL2L13 (abbreviated as B2L13) exhibited definitive pro-apoptotic activity. Previous studies have shown that overexpression of BCL2L13 in cell lines such as MCF-7 (breast cancer cells) [35], PC-3 (human prostate cancer cells) [36], and 293T [37] all upregulate apoptosis levels. Consistent with this, overexpression of BCL2L13 in Jurkat cells also increased the proportion of apoptotic cells, as validated by Western blot and flow cytometry analyses (Fig. 5D–F). Statistical analysis of BCL2L13 protein relative expression in bystander T cells across different groups showed significantly higher expression in the Omicron group than in both Mock and WT (ancestral strain) groups (Fig. 5B, Supplementary Fig. 7A). Furthermore, analysis of single-cell RNA (sc-RNA) sequencing data from bronchoalveolar lavage fluid (BALF) of COVID-19 patients revealed that BCL2L13 expression in T cells was higher in Omicron patients than in ancestral strain patients (Fig. 5C). This discovery provides a new molecular mechanism for the pathological feature of excessive T cell exhaustion following Omicron infection, suggesting that BCL2L13 may exacerbate T cell exhaustion via an “apoptosis amplifier” effect.

Given the differential levels of bystander T cell apoptosis mediated by different variants, we hypothesized that the specific upregulation of BCL2L13 in T cells following Omicron exposure is driven by cytokine secretion from infected epithelial cells. To test this, we performed transcriptomic sequencing on epithelial cells in the co-culture system and proteomic analysis on concentrated cell supernatants (Fig. 5G). Integration of upregulated genes and proteins across both omics datasets identified five proteins simultaneously elevated in epithelial cells and supernatants upon Omicron stimulation: GDF15, FCHSD1, GPNMB, HSF5, and SMAP2 (Fig. 5H). To validate the regulatory role of these candidate proteins in activating the downstream “apoptosis amplifier” BCL2L13, we added purified recombinant forms of the five proteins to T cell cultures and assessed BCL2L13 expression. Notably, GDF15 recombinant protein induced significant upregulation of BCL2L13 and increased T cell apoptosis (Fig. 5I). To further validate the role of the GDF15-BCL2L13 axis in driving high-intensity bystander T cell apoptosis, we knocked down BCL2L13 in T cells (Supplementary Fig 7B), Jurkat T cells in the co-culture system were pre-transfected with siBCL2L13-1, after which bystander T cell apoptosis was assessed. Results showed that while bystander T cell apoptosis was still induced, the Omicron-specific high-intensity apoptosis was abrogated, with no significant difference in apoptosis levels between the WT (ancestral strain) and Omicron groups (Fig. 5J, K).

### Elevated GDF-15 expression is associated with adverse prognosis in Omicron-positive COVID-19 patients

**Fig. 6:**
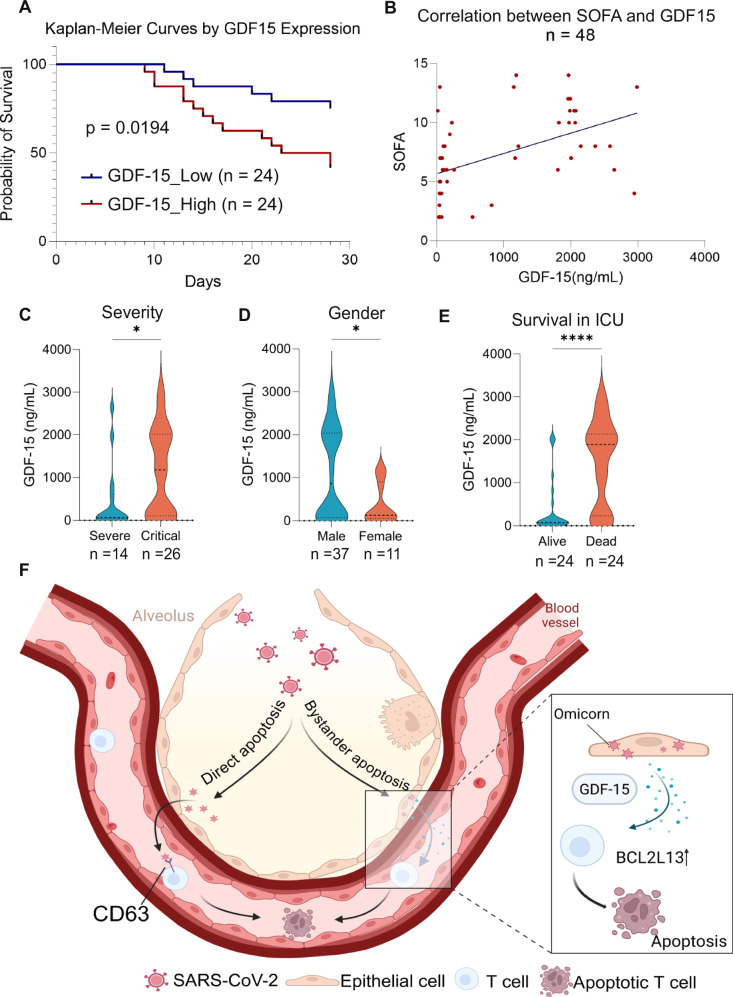
Elevated GDF-15 expression is associated with adverse prognosis in Omicron-positive COVID-19 patients. **A** Kaplan-Meier survival curve showing higher mortality in GDF-15 High group versus Low group in Omicron patients (*n* = 48). **B** Correlation analysis between plasma GDF-15 levels and SOFA scores in COVID-19 (Omicron) patients (*n* = 48). **C** Differential plasma GDF-15 expression between severe (*n* = 12) and critical (*n* = 26) Omicron-infected patients. **D** Differential plasma GDF-15 expression between male (*n* = 37) and female (*n* = 11) COVID-19 patients. **E** Differential plasma GDF-15 expression between ICU non-survivors (*n* = 24) and survivors (*n* = 24) of COVID-19. **F** Schematic diagram of SARS-CoV-2 infection-induced T cell apoptosis.

The correlation between GDF-15 and COVID-19 severity has been reported [38, 39]. We proposed that GDF-15 serves as an extracellular inducer of high-intensity T cell apoptosis in Omicron infection, which was further validated using clinical samples. We included plasma samples from 48 Omicron patients for ELISA assay and divided all samples into two groups based on the median GDF-15 expression level. The GDF-15 high group exhibited a higher mortality rate (Fig. 6A, Table 2), and plasma GDF15 levels positively correlated with SOFA scores (Fig. 6B). GDF15 levels were significantly higher in critically ill patients compared to severe cases, in ICU non-survivors versus survivors, and in male versus female patients (Fig. 6C–E). These findings provide a molecular rationale for the increased severe disease risk in male Omicron-infected individuals, as elevated GDF15 expression in males may contribute to enhanced T cell exhaustion. Notably, the GDF-15 High group exhibited significantly lower lymphocyte counts than the GDF-15 Low group, providing clinical evidence for the potential role of GDF-15 in T cell damage.

**Table 2:** Characteristics of COVID-19 patients with different GDF15 levels.

	All (*n* = 48)	GDF-15 High (*n* = 24)	GDF-15 Low (*n* = 24)	*p* value
**Demographics**				
Sex (females)^a^	11 (22.92)	5 (20.83)	6 (25.00)	0.7313^c^
Age (years)^b^	76 (70, 83)	76 (70, 84.25)	75.5 (34, 80.5)	0.4758^d^
BMI^b^	24.22 (22.47, 26.26)	24.22 (22.58, 25.42)	24.25 (16.14, 27.39)	0.5424^d^
**Comorbidities**				
Hypertension^a^	32 (66.67)	14 (58.33)	18 (75.0)	0.2207^c^
Diabetes^a^	22 (45.83)	10 (41.67)	12 (50.0)	0.5623^c^
Chronic kidney disease^a^	6 (12.5)	4 (16.67)	2 (8.33)	0.6662^e^
Coronary artery disease^a^	9 (18.75)	3 (12.5)	6 (25.0)	0.4614^e^
**COVID-19 severity**				
Mild/moderate^a^	8 (16.67)	4 (16.67)	4 (16.67)	>0.9999^e^
Severe^a^	14 (29.17)	4 (16.67)	10 (41.67)	0.0567^c^
Critical^a^	26 (54.17)	16 (66.67)	10 (41.67)	0.0822^c^
**Severity Score of illness**				
Apache II^b^	18 (12, 21.5)	19 (14.75, 25)	13.5 (8, 18.25)	0.0193^d^
SOFA score^b^	7 (4.25, 10)	9 (6.75, 11.25)	5.5 (2, 7.25)	0.0012^d^
**Vital Signs**				
Body temperature(°C)^b^	36.90 (36.5, 37.3)	37 (36.6, 37.5)	36.6 (36, 37)	0.0363^d^
Respiratory Rate (bpm)^b^	21 (16, 24.75)	21.5 (18.25, 26.25)	19 (12, 24)	0.2866^d^
Heart rate (bpm)^b^	91 (78, 106)	94.5 (85.25, 113.25)	83.5 (62, 104.25)	0.1033^d^
**Clinical Laboratory Results**				
Blood pH^b^	7.40 (7.37, 7.45)	7.40 (7.35, 7.40)	7.42 (7.20, 7.45)	0.1165^d^
Oxygen Saturation (SpO₂%)^b^	97 (92, 98)	95.5 (92, 97)	97 (87, 98)	0.1181^d^
PaO2^b^	69.90 (62.73, 83.48)	70.5 (60.48, 77.4)	69.9 (57.1, 82.73)	0.5489^d^
PaCO2^b^	37.15 (34.8, 41.95)	37.15 (34.8, 41.95)	34.1 (24, 38.33)	0.1077^d^
PaO2/FiO2^b^	139.5 (97.43, 202.6)	117.9 (90.17, 197.05)	154.15 (76, 191)	0.1433^d^
WBC (10⁹/L)^b^	8.28 (5.62, 9.98)	8.89 (5.63, 9.98)	7.10 (3.93, 9.80)	0.6753^d^
Neutrophils (10⁹/L)^b^	6.80 (4.67, 8.70)	7.94 (4.92, 8.70)	5.60 (2.35, 8.29)	0.4709^d^
Lymphocyte (10⁹/L)^b^	0.63 (0.37, 0.83)	0.47 (0.27, 0.70)	0.72 (0.27, 0.92)	0.0290^d^
**Respiratory support**				
Oxygen therapy^a^	16 (33.33)	2 (8.33)	4 (16.67)	0.6662^e^
HFNC^a^	23 (47.92)	2 (8.33)	6 (25.0)	0.2448^e^
Mechanical ventilation^a^	34 (70.83)	20 (83.33)	14 (58.33)	0.0567^c^
**LOS in ICU (days)**^**b**^	15 (10.75, 28)	21.5 (13.75, 29.5)	13.5 (5, 22.5)	0.0523^d^
**LOS in hospital (days)**^**b**^	20.5 (14, 32)	24 (17, 32.25)	16 (6, 22.5)	0.0219^d^
**ICU mortality**^a^	24 (50.00)	18 (75.00)	6 (25.00)	0.0005^c^
**28-day mortality**^a^	20 (41.67)	14 (58.33)	6 (25.00)	0.0192^c^

*HFNC* high-flow nasal cannula oxygen therapy, *LOS* length of stay.

^a^– Frequency (%).

^b^– Median (IQR).

^c^– Chi-square test.

^d^– Mann-Whiney U test.

^e^– Fisher’s exact test.

In summary, this discovery mechanistically explains the clinical observation of low T cell counts in severe Omicron infections by identifying the GDF15-BCL2L13 signaling axis as a key driver of amplified bystander T cell apoptosis. These findings for the first time link this axis to immune exhaustion, sex-specific outcomes, and prognostic differences in Omicron-infected patients, offering critical molecular markers for therapeutic intervention.

## Supplementary information

Supplementary material

WB_orginal data

FACs_original data

## Data Availability

The mass spectrometry proteomics data have been deposited to the ProteomeXchange Consortium (https://proteomecentral.proteomexchange.org) via the iProX partner repository with the dataset identifiers PXD071450 and PXD071622. The RNA-seq data have been deposited in the OMIX database under accession numbers OMIX015179 and OMIX013909. The single-cell transcriptomics data have been deposited in the Gene Expression Omnibus (GEO) under accession number GSE312913. The anonymized dataset analyzed in this study can be made available upon reasonable request. Requests must be accompanied by a detailed research protocol and analysis plan, and must have appropriate institutional approvals, including data transfer agreements, in place prior to access. Requests should be formally addressed to Professor Haibo Qiu, Head of the Jiangsu Provincial Key Laboratory of Critical Care Medicine (haiboq2000@163.com).
