# Supplementary material for: Variant-divergent death: Omicron intensifies bystander T-cell apoptosis via GDF15–BCL2L13
Source: Cell Death Discov. 2026 Mar 28;12:201. doi: 10.1038/s41420-026-03079-x (PMC13150034; doi:10.1038/s41420-026-03079-x)
Supplement: Supplementary file 1 — Supplementary material [file 41420_2026_3079_MOESM1_ESM.pdf]

Supplementary

Conserved Host Hijacking Mechanisms of SARS-CoV-2 Variants: Molecular Reprogramming Drives T Lymphocyte Dysfunction

In this study, Jurkat T cells were infected with ancestral SARS-CoV-2 and Omicron (MOI = 0.05), and three groups of RNA were collected at three time points (0 h, 24 h, 48 h) for RNA-seq (Supp Fig 4A), to horizontally compare differences between variants and vertically compare T cell infection dynamics over time. Principal component analysis (PCA) of RNA-seq data showed that scatter plots of the two variants had smaller distances after dimensionality reduction, while those of different time points were more dispersed, indicating that direct infection of T cells by the two COVID-19 variants at different periods exerted no significant difference, whereas T cell changes at different infection times were more distinct (Supp Fig 4B). To further validate this, weighted gene co-expression network analysis (WGCNA) was performed on transcriptomic data. Hierarchical clustering of all genes identified 17 gene modules (Supp Fig 4C, 4D), with ancestral SARS-CoV-2 and Omicron showing similar expression profiles—i.e., the same gene module was concurrently upregulated or downregulated at the same time point post-infection (Supp Fig 4E). Proteomic analysis using the same experimental groups showed PCA results similar to RNA-seq (Supp Fig 4F), confirming minimal cellular changes induced by different variants.

Ribosome Biogenesis and Protein Translation Pathway Alterations Mediate Functional Dysfunction of Bystander T Cells

To better understand the molecular changes in bystander T cells during apoptosis, proteomic analysis of bystander T cells was performed, identifying significantly differentially expressed proteins (DEPs) in wild-type (WT) and Omicron infection groups compared to the control group. GO biological process (BP) enrichment analysis showed that WT DEPs were primarily enriched in cytoplasmic translation, ribosome biogenesis, rRNA metabolic process, regulation of post-translational protein modification, and ncRNA processing (Supp Fig 6A). Notably, Omicron DEPs exhibited highly similar enrichment directions to WT, but with a significantly increased number of enriched proteins in cytoplasmic translation and rRNA metabolic processes (Supp Fig 6B), suggesting that the Omicron strain may exert stronger interference with host cell translation and RNA metabolism-related pathways. Further KEGG enrichment analysis showed that WT DEPs were mainly enriched in ribosome, COVID-19, and protein processing in endoplasmic reticulum pathways (Supp Fig 6C). The Omicron group also showed significant enrichment in ribosome and COVID-19 pathways, but with stronger enrichment significance (lower p-values) (Supp Fig 6D).

These results indicate that both ancestral and Omicron strains induce functional dysfunction of bystander T cells by targeting the ribosome biogenesis and protein translation systems. The common enrichment of ribosomal pathways may reflect a conserved viral strategy of hijacking host translation machinery to support self-

replication. Additionally, enrichment in the COVID-19 pathway suggests that virus infection-related immune signals (such as cytokine storms or antigen presentation) may indirectly exacerbate metabolic stress in bystander T cells.

Supplement Videos. Dynamic microscopy imaging of SARS-CoV-2 pseudovirus entry mediated by CD63-overexpressing cells.

Supplement Figure 1. Heterogeneous, Lower-Tail Impact of Omicron on Lymphocyte Counts (Overlap-Weighted Analyses). (A) Overlap-weighted empirical cumulative distribution functions (ECDFs) of lymphocyte counts by variant. ATO (overlap) weights from a propensity-score model (covariates: age [restricted cubic splines], sex, hypertension, diabetes, chronic kidney disease, coronary artery disease, malignancies, COPD, cirrhosis) were applied prior to computing ECDFs. The dashed vertical line marks the reference threshold (1.0 × 10^9/L). A higher curve at low values indicates a greater cumulative probability of low lymphocyte counts under Omicron compared with the ancestral strain. (B) Overlap-weighted quantile treatment effect (QTE) curve showing quantile treatment ratios (QTR = Omicron/Ancestral) across τ = 0.05 – 0.50. QTR < 1 at the lower tail (τ ≈ 0.05 – 0.30) indicates lower lymphocyte levels under Omicron at low quantiles, with attenuation toward the median. This tail-focused pattern aligns with stronger bystander apoptosis.

Supplement Figure 2. Low pH promotes syncytia formation and CD63-mediated clathrin endocytosis. (A, B) The CD63 overexpression cells was successfully

constructed as detected by flow cytometry. (C) The CD63 overexpression Jurkat cells was successfully constructed. (D, E) Comparison of molecular docking affinity between CD63 and SARS-CoV-2 Spike (HIV-gp41 as positive control). (F) Schematic diagram of the large extracellular loop (LEL) of the CD63 molecule. (G) Low pH environment (treated with pH 4.0 PBS for 30 min followed by medium replacement) promotes increased fusion and syncytia formation in CD63-overexpressing cells. (H, I) Low pH environment enhances CD63-mediated viral infection. (J, K) Screening of endocytic pathway inhibitors for endocytic modes dependent on ancestral SARS-CoV-2 and Omicron infection; Dynasor (100 μM, pre-treated for 60 min), Nystatin (50 μM, pre-treated for 90 min), Blebbistatin (100 μM, pre-treated for 120 min).

Supplement Figure 3. Apoptosis-related genes exhibit the highest proportion of changes in COVID-19 patients. (A) Intersection analysis of differentially expressed genes (DEGs) between ancestral SARS-CoV-2-infected individuals and healthy with cell death-related gene sets, showing the highest proportion of apoptosis-related genes. (B) Intersection analysis of DEGs between Omicron-infected individuals and healthy with cell death-related gene sets, with the highest proportion of apoptosis-related genes.

Supplement Figure 4. Molecular characterization of T cells infected with ancestral SARS-CoV-2 and Omicron. (A) Schematic of experimental groups (n=3). (B) Dimensionality reduction plot of RNA-seq data via PCA. (C) Schematic of gene module partitioning by WGCNA. (D) Correlation heatmap of gene modules from WGCNA. (E) Correlation between gene modules and experimental groups. The y-axis represents gene modules, and the x-axis represents experimental groups. Blue indicates

module downregulation in the group, red indicates upregulation, with darker colors reflecting greater fold changes. Values within each cell denote p-values, where smaller values indicate more significant statistical differences. (F) Dimensionality reduction plot of proteome data via PCA.

Supplement Figure 5. Single-cell data reveals epithelial-immune regulation. (A) tSNE dimensionality reduction of single-cell data from bronchoalveolar lavage fluid. (B) Display of communication counts between different cell subsets. (C) Display of communication intensity between different cell subsets.

Supplement Figure 6. Proteomic signatures and pathway enrichment analysis of bystander T cell apoptosis. (A) GO analysis (biological process) of differentially expressed proteins in bystander T cells induced by the ancestral strain compared to the control group. (B) GO analysis (biological process) of differentially expressed proteins in bystander T cells induced by Omicron compared to the control group. (C) KEGG pathway enrichment analysis of differentially expressed proteins in bystander T cells induced by the ancestral strain compared to the control group. (D) KEGG pathway enrichment analysis of differentially expressed proteins in bystander T cells induced by Omicron compared to the control group.

Supplement Figure 7. Validation of BCL2L13 expression levels. (A) Proteomic analysis of relative expression levels of BCL2L13 in bystander T cells. (B) Validation of BCL2L13 knockdown.

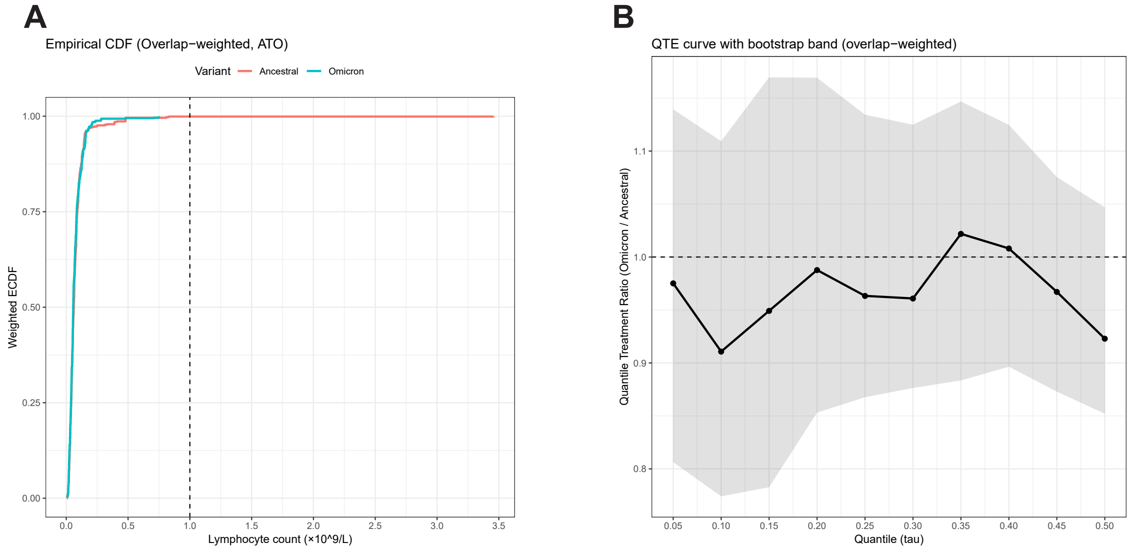

Supplement Figure 1. Heterogeneous, Lower-Tail Impact of Omicron on Lymphocyte Counts (Overlap-Weighted Analyses). (A) Overlap-weighted empirical cumulative distribution functions (ECDFs) of lymphocyte counts by variant. ATO (overlap) weights from a propensity-score model (covariates: age [restricted cubic splines], sex, hypertension, diabetes, chronic kidney disease, coronary artery disease, malignancies, COPD, cirrhosis) were applied prior to computing ECDFs. The dashed vertical line marks the reference threshold (1.0 × 10^9/L). A higher curve at low values indicates a greater cumulative probability of low lymphocyte counts under Omicron compared with the ancestral strain. (B) Overlap-weighted quantile treatment effect (QTE) curve showing quantile treatment ratios (QTR = Omicron/Ancestral) across $\tau = 0.05 – 0.50$. QTR < 1 at the lower tail ($\tau \approx 0.05 – 0.30$) indicates lower lymphocyte levels under Omicron at low quantiles, with attenuation toward the median. This tail-focused pattern aligns with stronger bystander apoptosis.

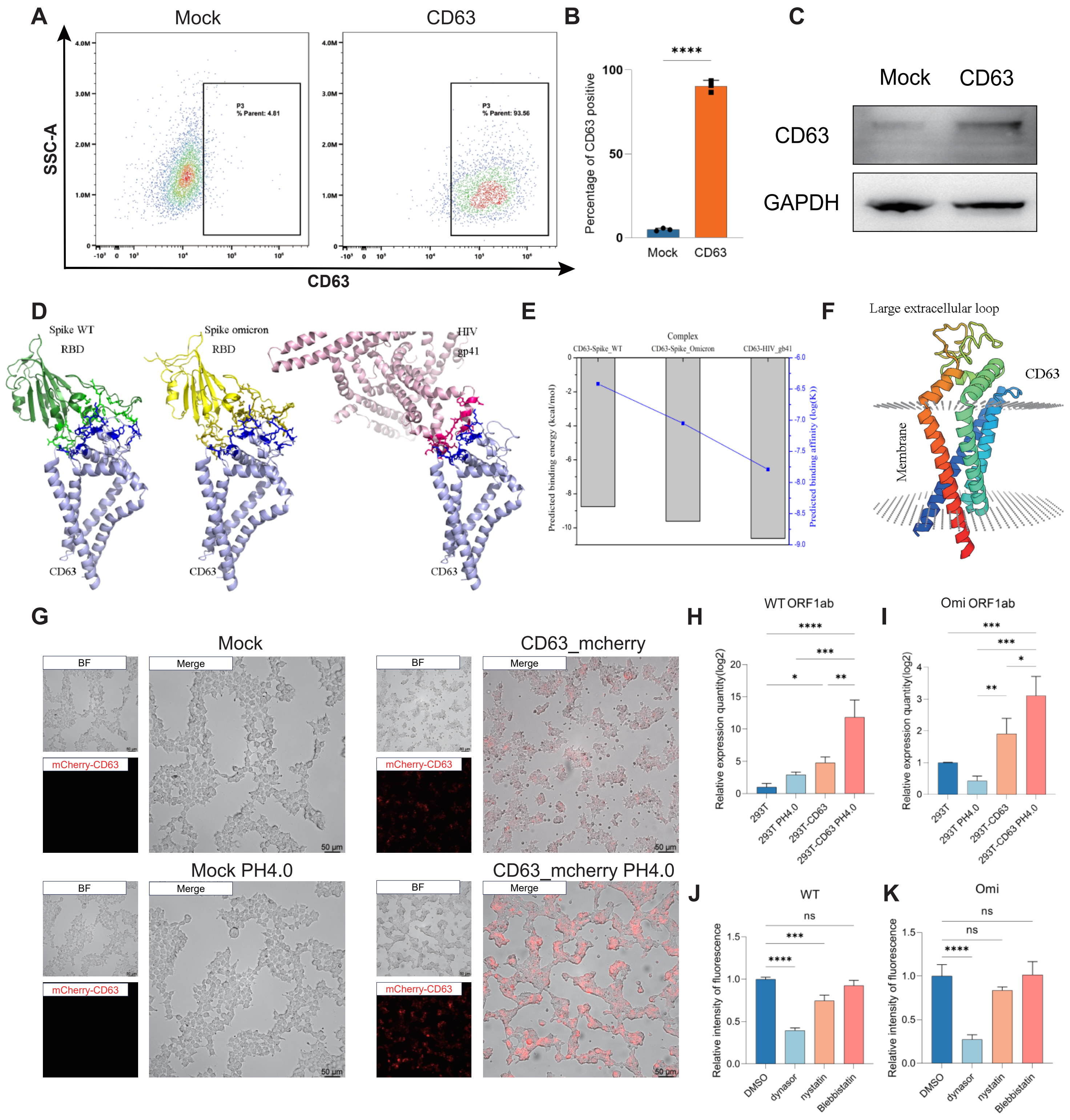

Supplement Figure 2. Low pH promotes syncytia formation and CD63-mediated clathrin endocytosis. (A, B) The CD63 overexpression cells was successfully constructed as detected by flow cytometry. (C) The CD63 overexpression Jurkat cells was successfully constructed. (D, E) Comparison of molecular docking affinity between CD63 and SARS-CoV-2 Spike (HIV-gp41 as positive control). (F) Schematic diagram of the large extracellular loop (LEL) of the CD63 molecule. (G) Low pH environment (treated with pH 4.0 PBS for 30 min followed by medium replacement) promotes increased fusion and syncytia formation in CD63-overexpressing cells. (H, I) Low pH environment enhances CD63-mediated viral infection. (J, K) Screening of endocytic pathway inhibitors for endocytic modes dependent on ancestral SARS-CoV-2 and Omicron infection; Dynasor (100 μM, pre-treated for 60 min), Nystatin (50 μM, pre-treated for 90 min), Blebbistatin (100 μM, pre-treated for 120 min).

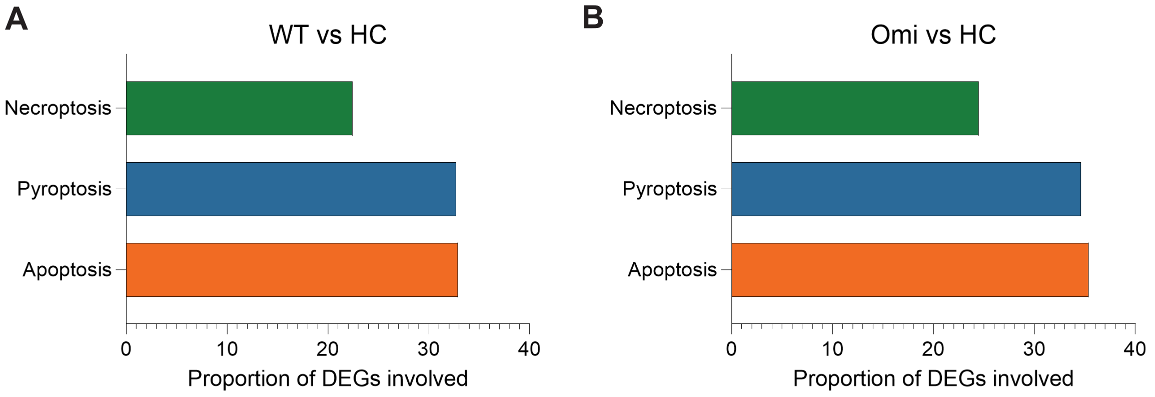

Supplement Figure 3. Apoptosis-related genes exhibit the highest proportion of changes in COVID-19 patients. (A) Intersection analysis of differentially expressed genes (DEGs) between ancestral SARS-CoV-2-infected individuals and healthy with cell death-related gene sets, showing the highest proportion of apoptosis-related genes. (B) Intersection analysis of DEGs between Omicron-infected individuals and healthy with cell death-related gene sets, with the highest proportion of apoptosis-related genes.

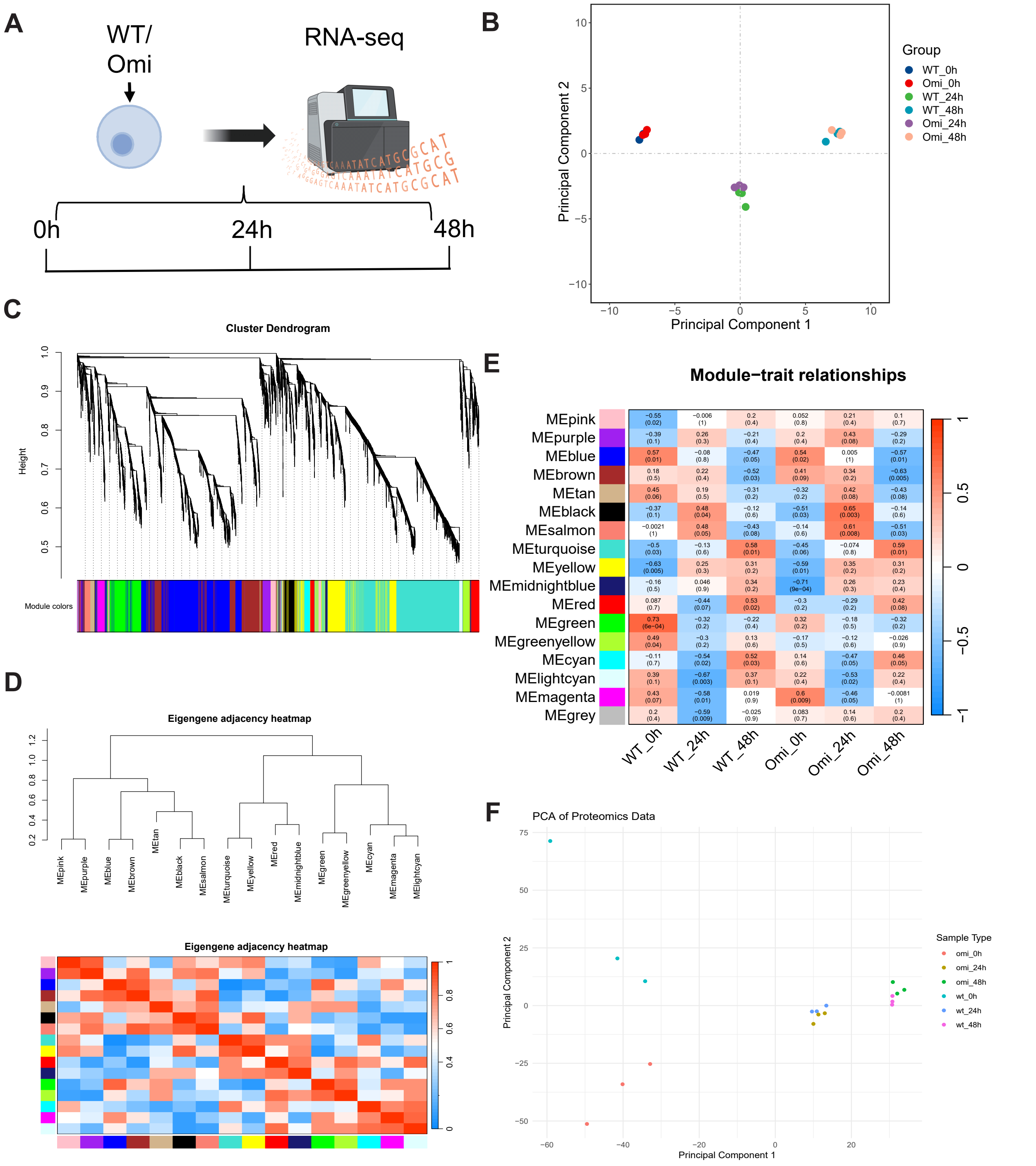

Supplement Figure 4. Molecular characterization of T cells infected with ancestral SARS-CoV-2 and Omicron. (A) Schematic of experimental groups (n=3). (B) Dimensionality reduction plot of RNA-seq data via PCA. (C) Schematic of gene module partitioning by WGCNA. (D) Correlation heatmap of gene modules from WGCNA. (E) Correlation between gene modules and experimental groups. The y-axis represents gene modules, and the x-axis represents experimental groups. Blue indicates module downregulation in the group, red indicates upregulation, with darker colors reflecting greater fold changes. Values within each cell denote p-values, where smaller values indicate more significant statistical differences. (F) Dimensionality reduction plot of proteome data via PCA.

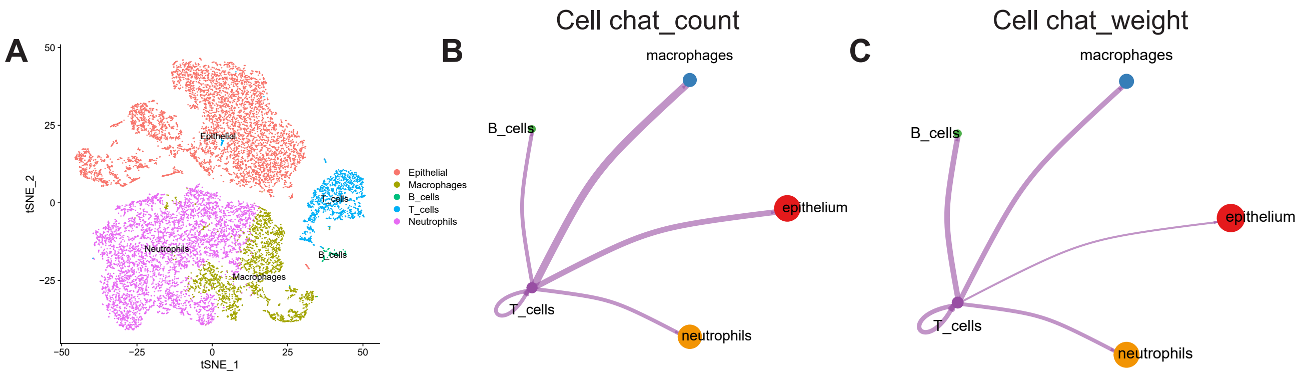

Supplement Figure 5. Single-cell data reveals epithelial-immune regulation. (A) tSNE dimensionality reduction of single-cell data from bronchoalveolar lavage fluid. (B) Display of communication counts between different cell subsets. (C) Display of communication intensity between different cell subsets.

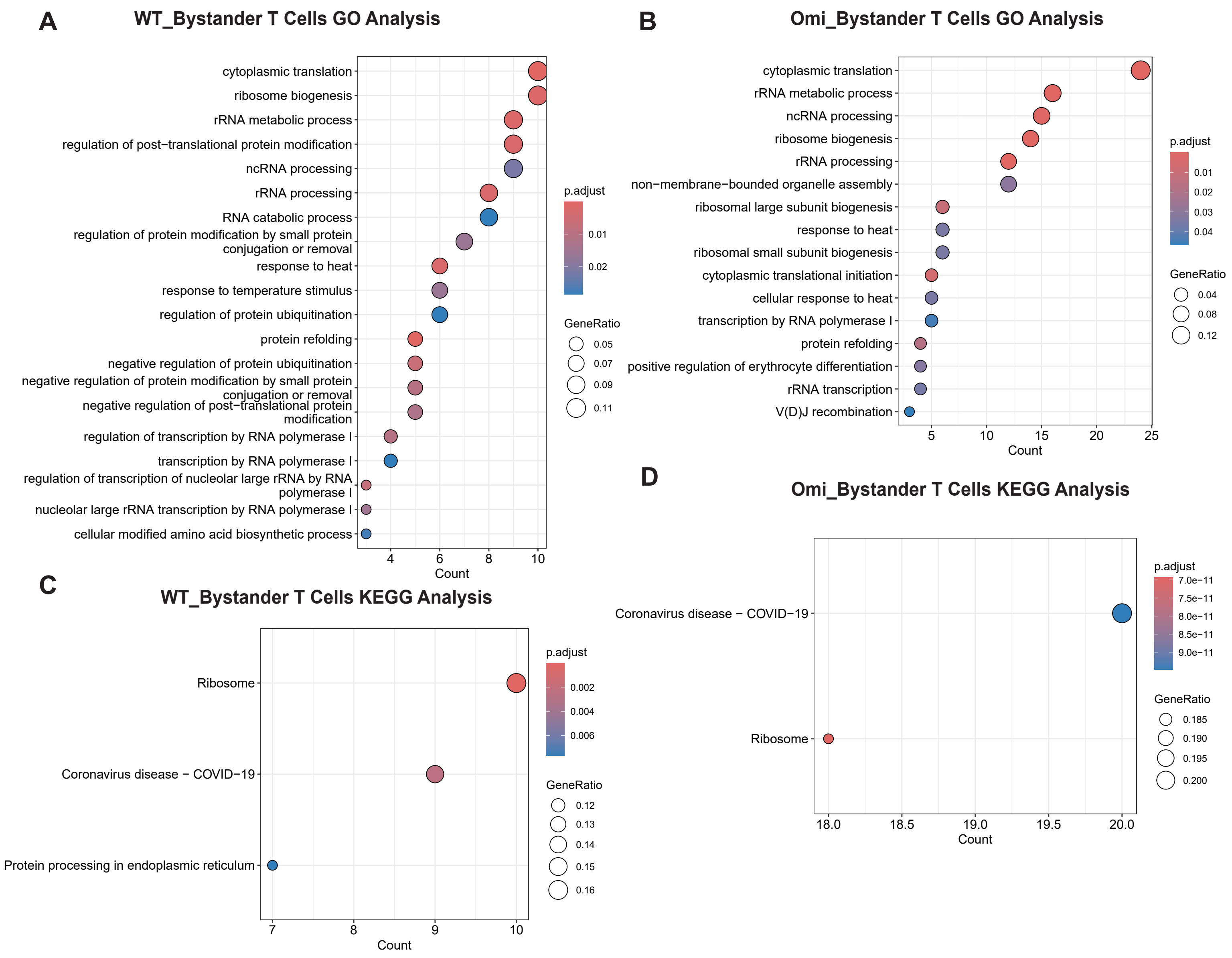

Supplement Figure 6. Proteomic signatures and pathway enrichment analysis of bystander T cell apoptosis. (A) GO analysis (biological process) of differentially expressed proteins in bystander T cells induced by the ancestral strain compared to the control group. (B) GO analysis (biological process) of differentially expressed proteins in bystander T cells induced by Omicron compared to the control group. (C) KEGG pathway enrichment analysis of differentially expressed proteins in bystander T cells induced by the ancestral strain compared to the control group. (D) KEGG pathway enrichment analysis of differentially expressed proteins in bystander T cells induced by Omicron compared to the control group

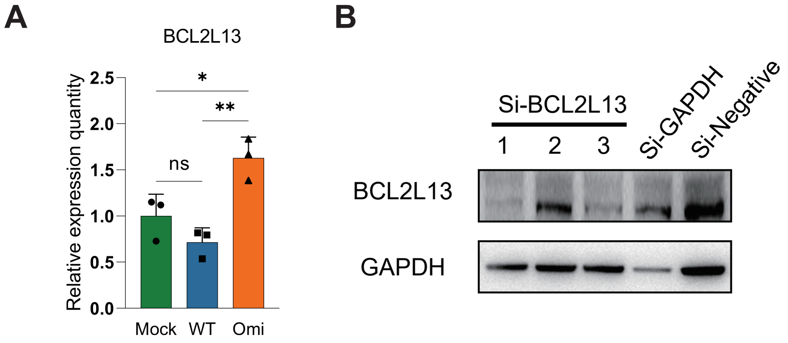

Supplement Figure 7. Validation of BCL2L13 expression levels. (A) Proteomic analysis of relative expression levels of BCL2L13 in bystander T cells. (B) Validation of BCL2L13 knockdown.

Supplement Table 1. Top 10 Differentially Expressed Proteins in Bystander T Cells (Omicron vs WT)

Rank	Alias used in manuscript	Gene symbol (HGNC)	Protein name	Brief function
1	RBM6	RBM6	RNA binding motif protein 6	RNA-binding protein
2	RT25	MRPS25	Mitochondrial ribosomal protein S25	Mitochondrial small ribosomal subunit
3	BC2L13	BCL2L13	BCL2 like 13	Pro-apoptotic BCL2 family member
4	EIPR1	EIPR1	EARP and GARP complex interacting protein 1	Endosome recycling
5	RM46	MRPL46	Mitochondrial ribosomal protein L46	Mitochondrial large ribosomal subunit
6	WDR46	WDR46	WD repeat domain 46	Nucleolar scaffold component
7	DGC14	DGCR14	DiGeorge syndrome critical region gene 14 (ESS-2 homolog)	Pre-mRNA splicing factor
8	MBOA7	MBOAT7	Membrane bound O-acyltransferase domain containing 7	Lysophospholipid acyltransferase 7
9	CLP1L	CLPTM1L	CLPTM1 like	Associated with post-translational processes
10	ERF3B	GSPT2 (eRF3b)	G1 to S phase transition 2 / Eukaryotic peptide chain release factor subunit 3b	Translation termination GTPase
