# Supplementary material for: Variant-divergent death: Omicron intensifies bystander T-cell apoptosis via GDF15–BCL2L13
Source: Cell Death Discov. 2026 Mar 28;12:201. doi: 10.1038/s41420-026-03079-x (PMC13150034; doi:10.1038/s41420-026-03079-x)
Supplement: Supplementary file 2 — WB_orginal data [file 41420_2026_3079_MOESM2_ESM.pdf]

Unedited original gel diagram for Figure2E, 2F WB was conducted to evaluate CD63, Flag protein levels

Fig2E

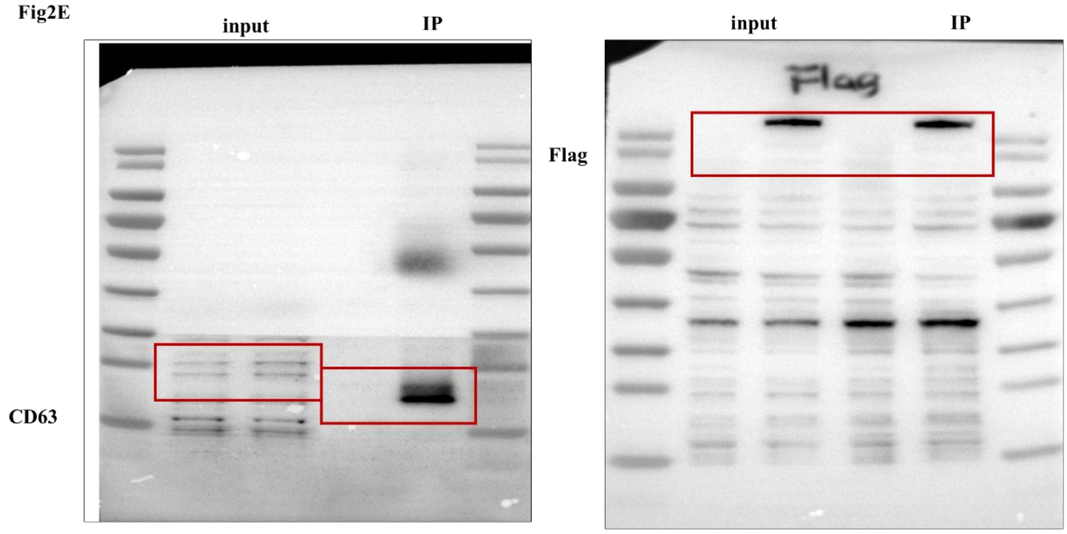

Fig2F

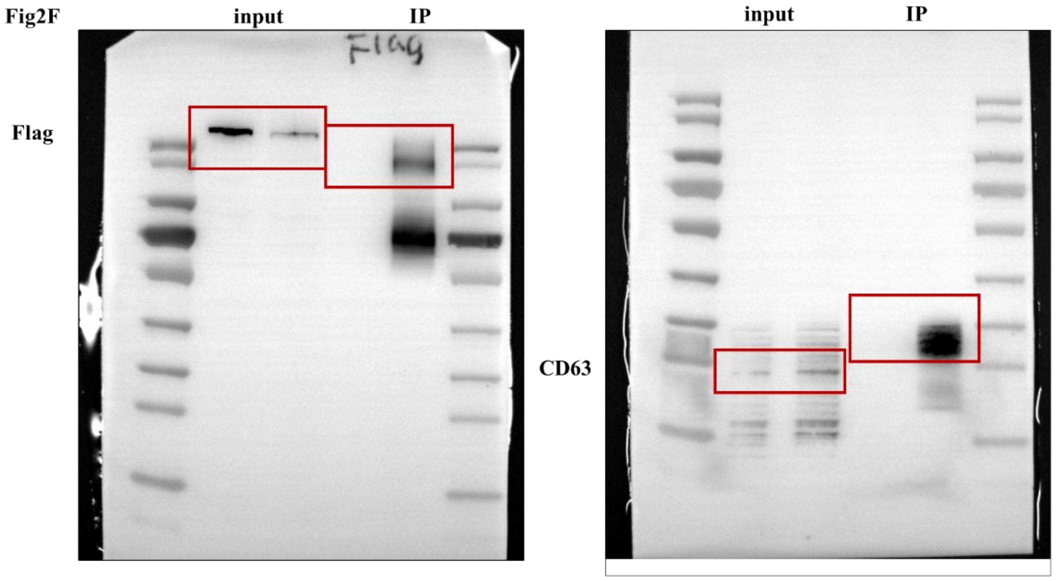

Unedited original gel diagram for Figure2G, 2H WB was conducted to evaluate CD63, His protein levels

Fig2G

input **IP**

CD63

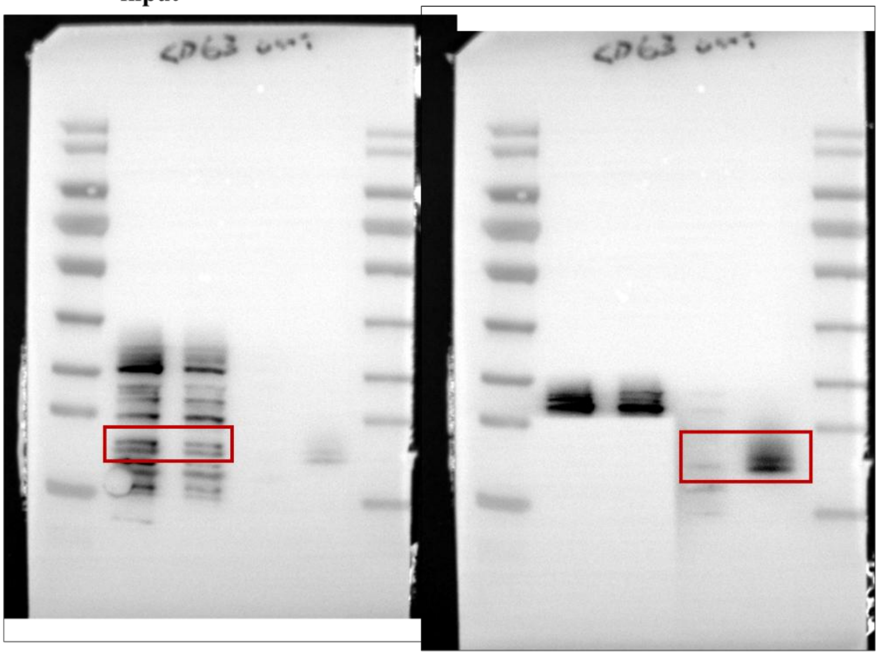

Fig2G

input **IP**

His

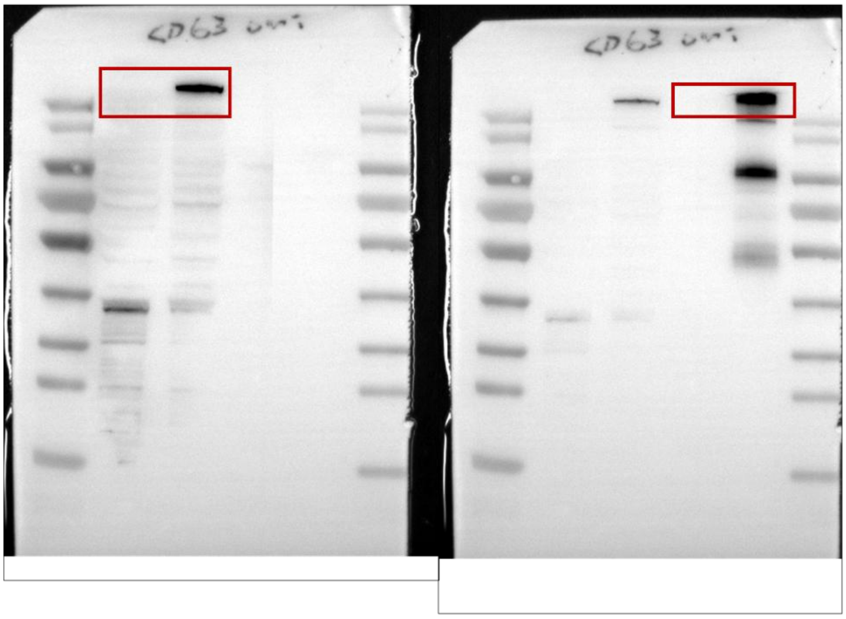

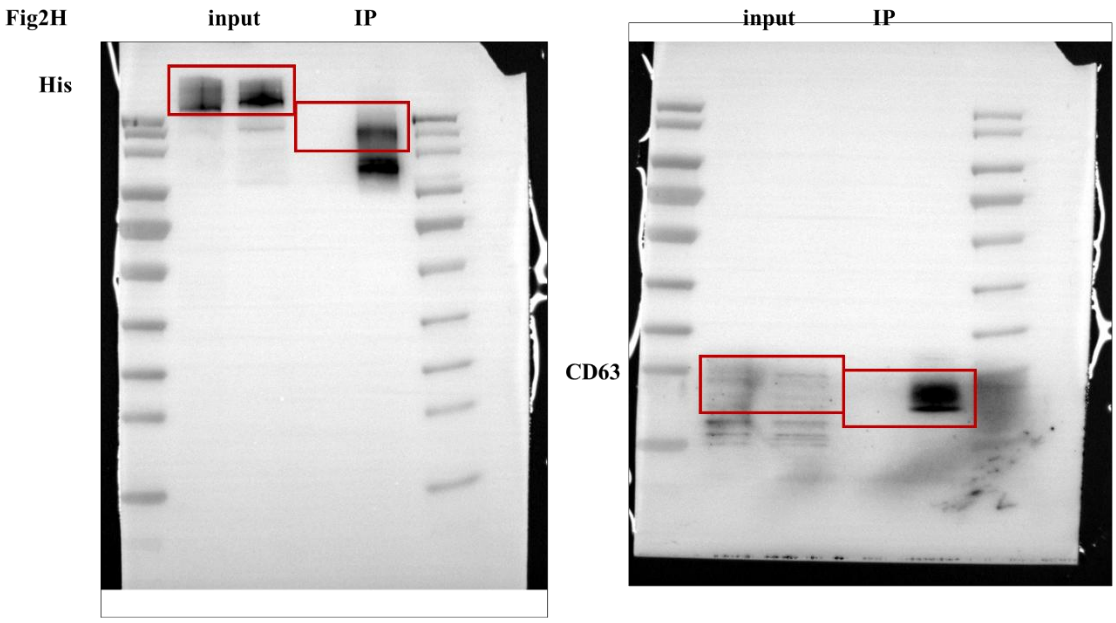
Fig2H
input
IP
His
input
IP
CD63

Unedited original gel diagram for Figure3F WB was conducted to evaluate N, caspase-3, cleaved-caspase-3, GAPDH protein levels

Fig3F

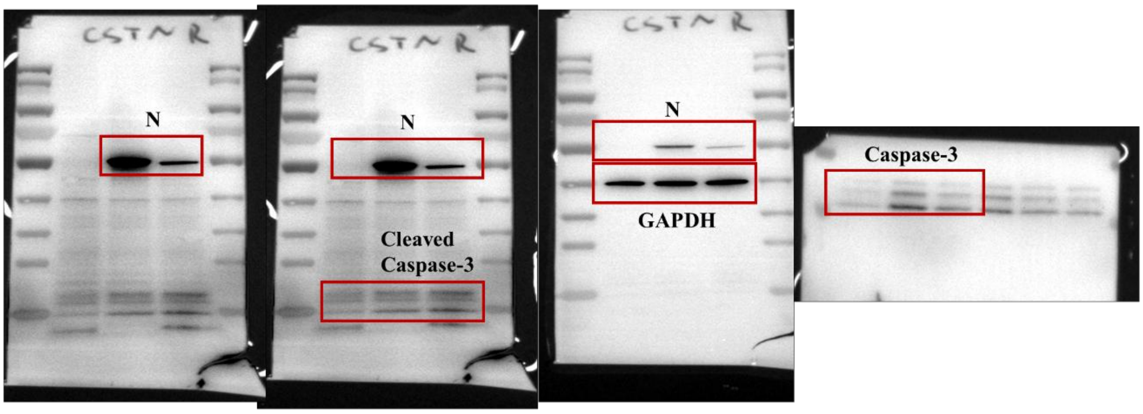

Unedited original gel diagram for Figure3J WB was conducted to evaluate GSDMD, N-GSDMD, GAPDH, MLKL, p-MLKL protein levels

Fig3J

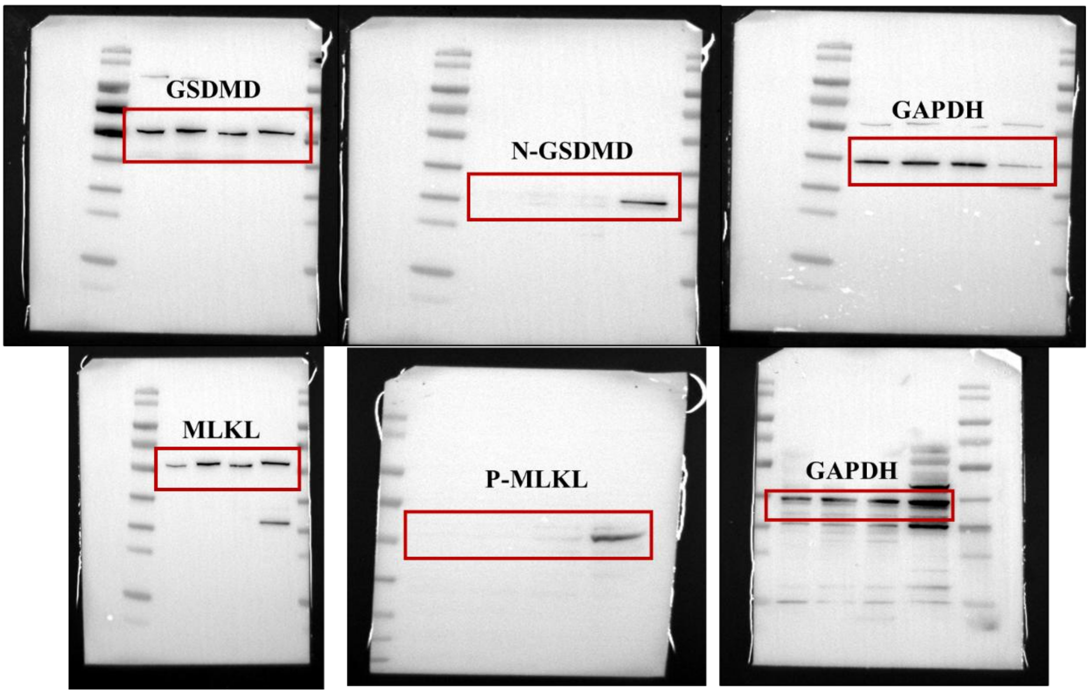

Unedited original gel diagram for Figure5B WB was conducted to evaluate BCL2L13, GAPDH protein levels

Fig5B

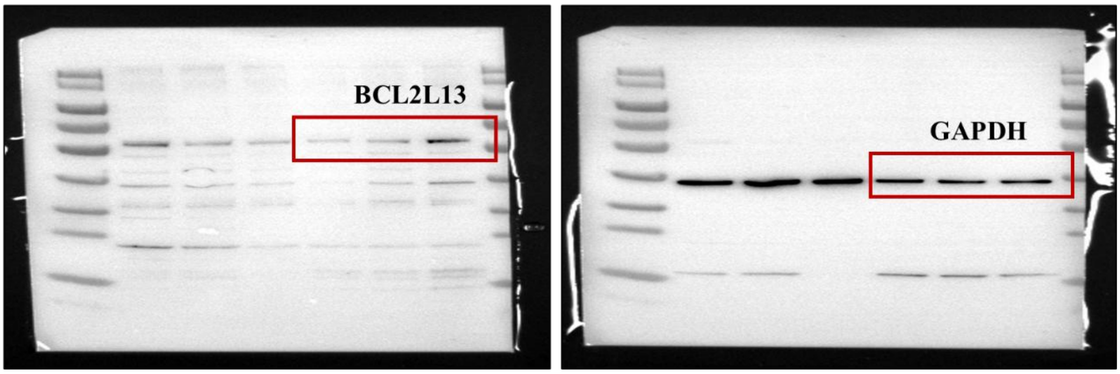

Unedited original gel diagram for Figure5D WB was conducted to evaluate BCL2L13, caspase-3, cleaved-caspase-3, GAPDH protein levels

Because BCL2L13 is a mitochondrial transmembrane protein with potential post-translational modifications and splice isoforms, Western blots with commercial antibodies may show multiple bands[1–3]; we verified by siRNA knockdown that the specific major band in our samples is ~55 kDa.

Fig5D

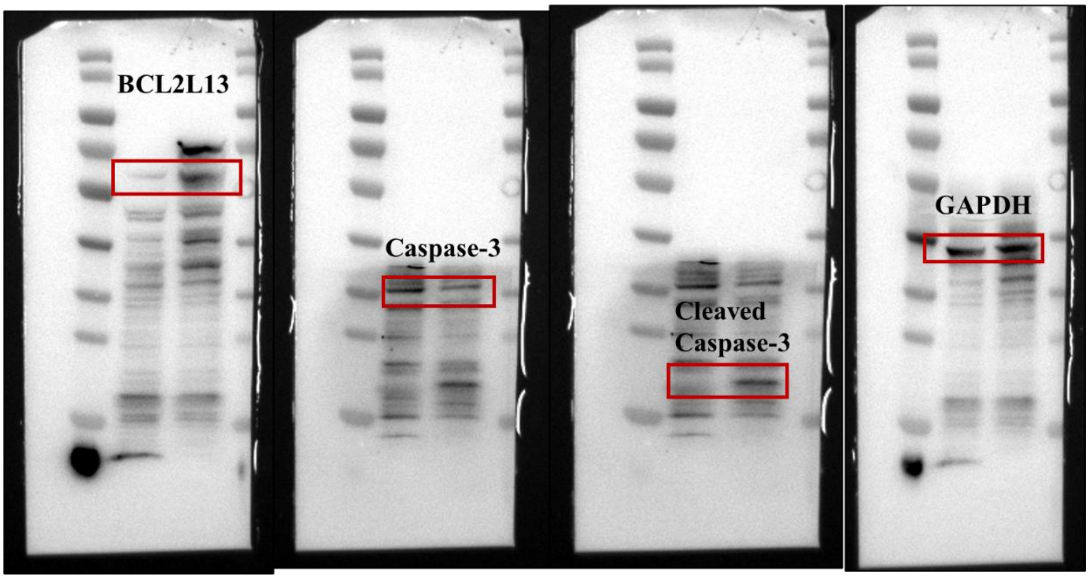

Unedited original gel diagram for Figure5I WB was conducted to evaluate BCL2L13, cleaved-caspase-3, GAPDH protein levels

Because BCL2L13 is a mitochondrial transmembrane protein with potential post-translational modifications and splice isoforms, Western blots with commercial antibodies may show multiple bands[1–3]; we verified by siRNA knockdown that the specific major band in our samples is ~55 kDa.

Fig5I

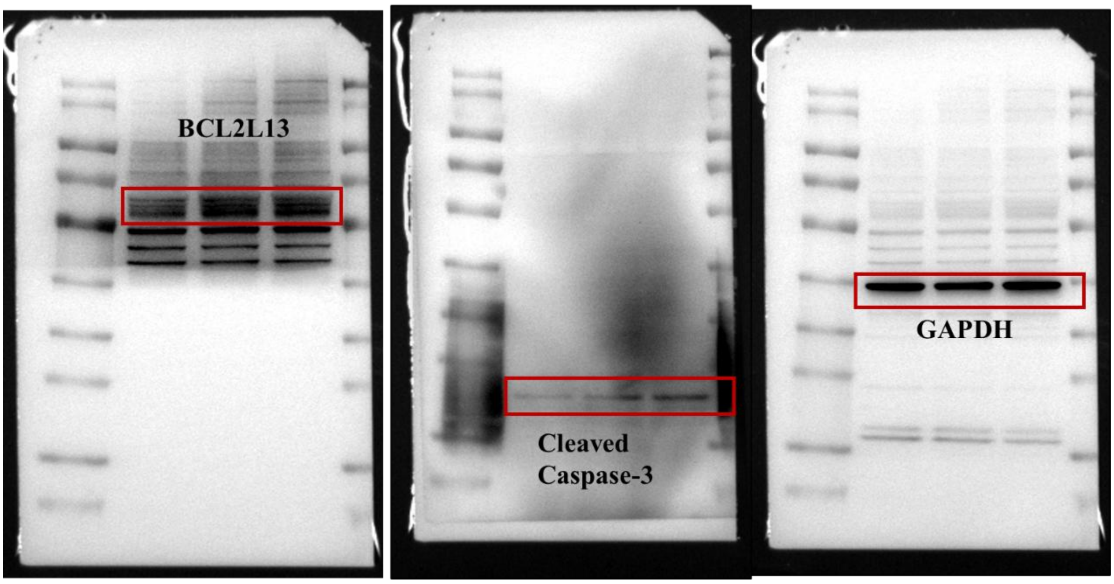

Unedited original gel diagram for Figure7B WB was conducted to evaluate BCL2L13, GAPDH protein levels

Because BCL2L13 is a mitochondrial transmembrane protein with potential post-translational modifications and splice isoforms, Western blots with commercial antibodies may show multiple bands[1–3]; we verified by siRNA knockdown that the specific major band in our samples is ~55 kDa.

Supp Fig7B

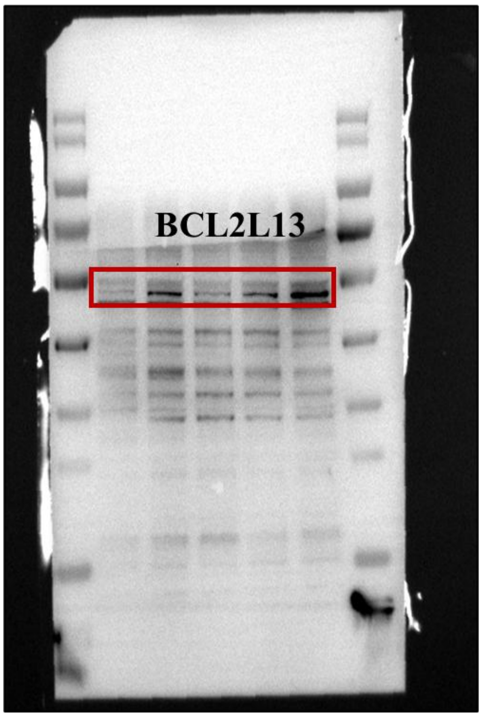

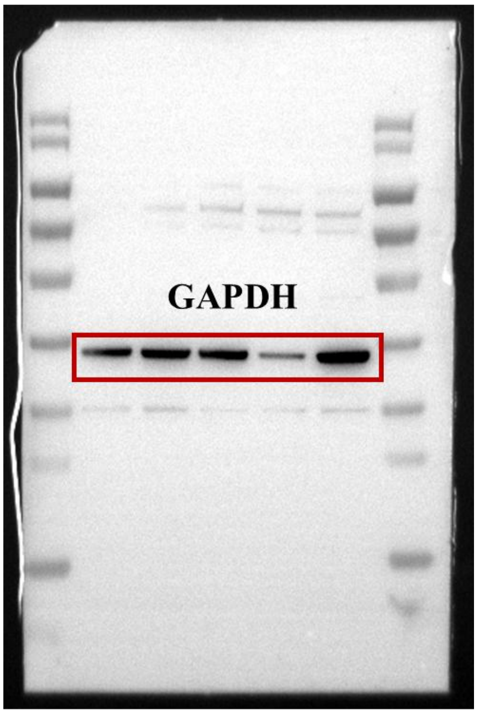

Reference

1. Kremer, L. S. *et al.* A role for BCL2L13 and autophagy in germline purifying selection of mtDNA. *PLoS Genet* **19**, e1010573 (2023).

2. Meng, F. *et al.* Down-regulation of BCL2L13 renders poor prognosis in clear cell and papillary renal cell carcinoma. *Cancer Cell Int* **21**, 332 (2021).

3. Kataoka, T. *et al.* Bcl-rambo, a novel Bcl-2 homologue that induces apoptosis via its unique C-terminal extension. *J Biol Chem* **276**, 19548–19554 (2001).
